# Supplementary material for: Interceptive Treatment with Invisalign® First in Moderate and Severe Cases: A Case Series
Source: Children (Basel). 2022 Aug 5;9(8):1176. doi: 10.3390/children9081176 (PMC9406487; doi:10.3390/children9081176)

Supplementary Figure S1. Twenty-three interceptive treatments cases with the highest complexity degree problem selected from the sample - A/D/G/J before treatment; B/E/H/K predicted using ClinCheck® simulation; and C/F/I/L at the end of the 18 months, according to the objectives to be achieved. Case 1: Posterior crossbite; Space recovery - severe (3). Case 2: Open bite; Crowding - severe (3). Case 3: Molar sagittal malocclusion II - severe (3). Case 4: Space recovery - Severe (2). Case 5: Open bite - severe (3). Case 6: Molar sagittal malocclusion II; Midline discrepancy - Severe (2). Case 7: Molar sagittal malocclusion II - moderate (2). Case 8: Crowding - Severe (2). Case 9: Midline discrepancy - severe (3). Case 10: Space recovery; Molar sagittal malocclusion II; Crowding - severe (3). Case 11: Midline discrepancy; Molar sagittal malocclusion II - severe (3). Case 12: Space recovery; Molar sagittal malocclusion III - severe (3). Case 13: Midline discrepancy - severe (3). Case 14: Molar sagittal malocclusion II - moderate (2). Case 15: Midline discrepancy - moderate (3). Case 16: Midline discrepancy; Posterior crossbite; Dentoalveolar expansion - severe (3). Case 17: Crowding - moderate (3). Case 18: Crowding; Midline discrepancy; Space recovery - severe (3). Case 19: Midline discrepancy - severe (3). Case 20: Open bite - severe (3). Case 21: Molar sagittal malocclusion II - severe (3). Case 22: Posterior crossbite - moderate (2). Case 23: Crowding; Midline discrepancy; Space recovery; Dentoalveolar expansion; Molar sagittal malocclusion II - severe (3).

Case 1: Severe - **<u>SR</u>**; PCB; MD; Cr; Transversal skeletal problem.

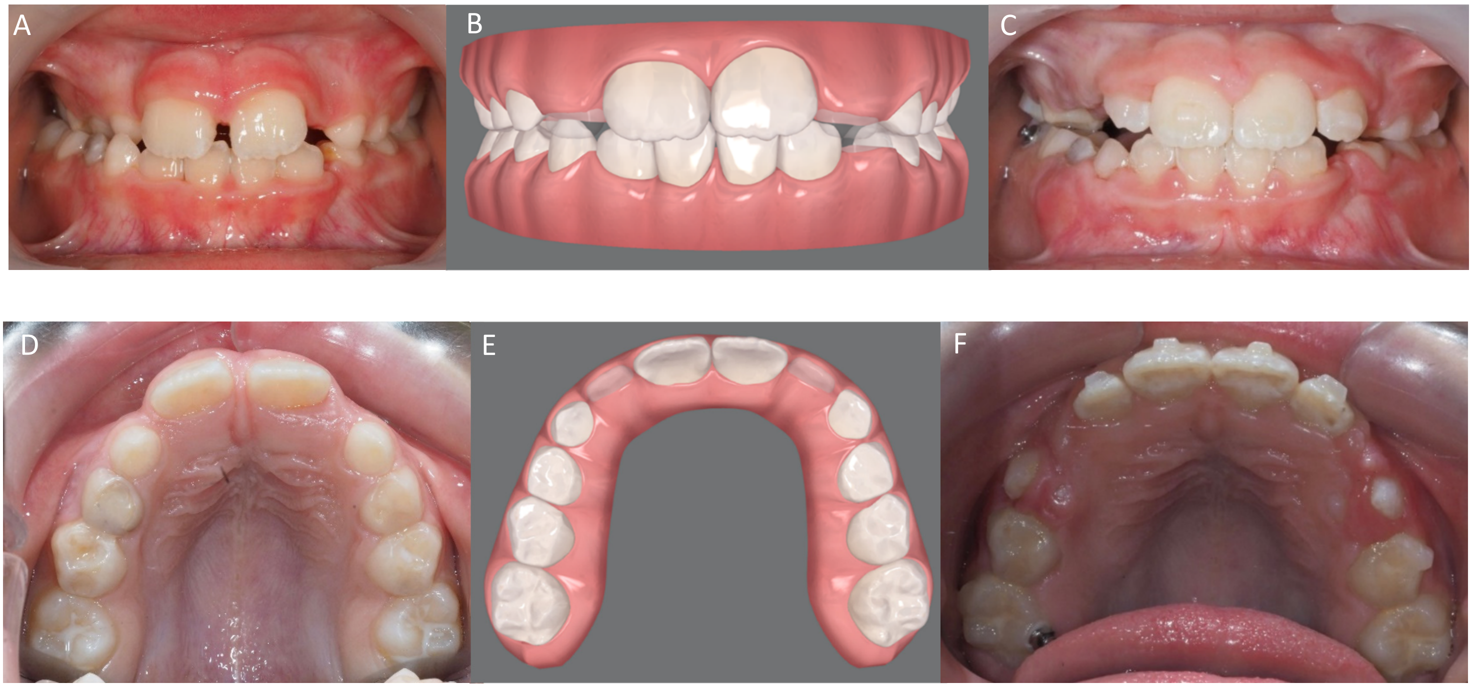

Case 2: Severe – OB; Vertical and sagittal skeletal problem.

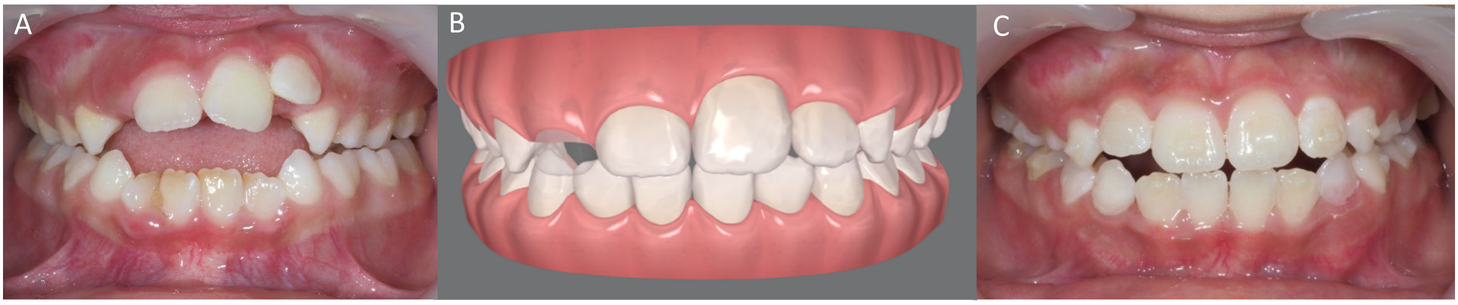

Case 3: Severe – **MC**; Sagittal skeletal problem.

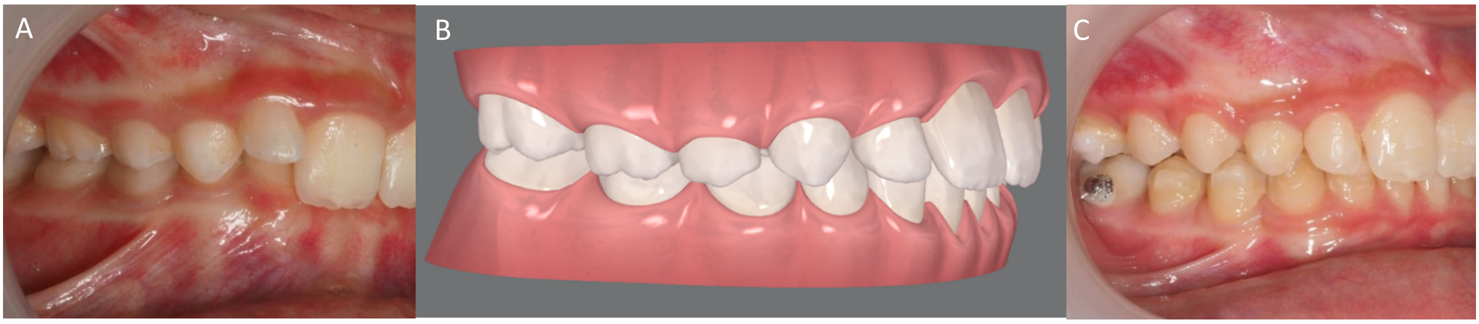

Case 4: Severe – **CR**; Sagittal skeletal problem.

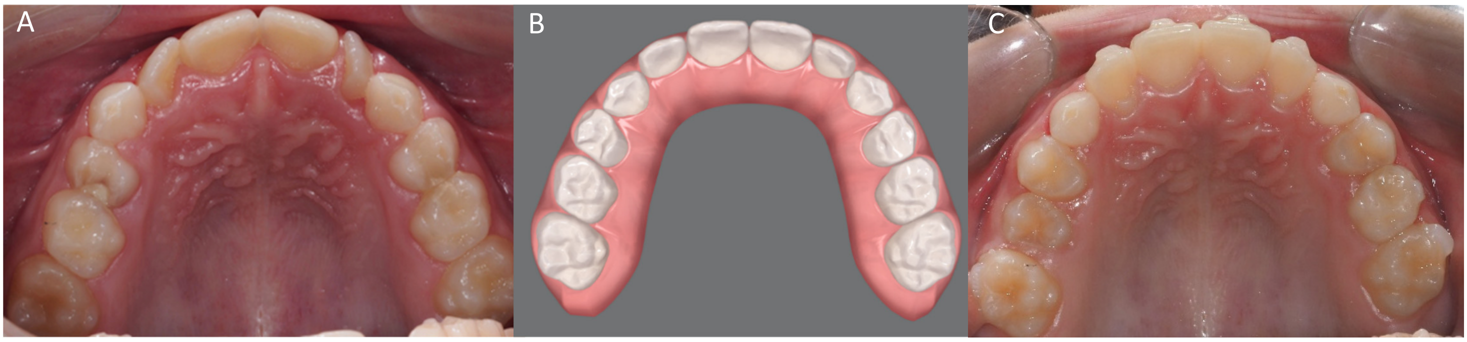

Case 5: Severe – **OB**; Vertical and transversal skeletal problem.

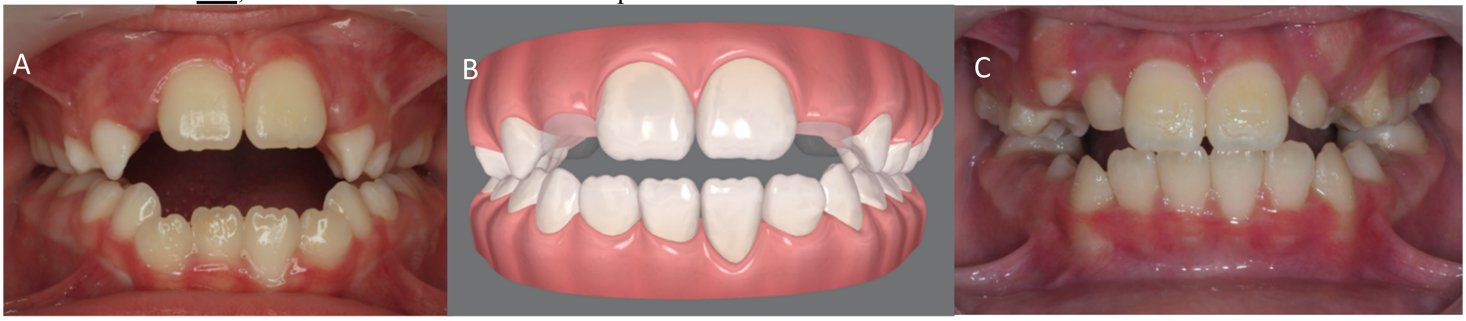

Case 6: Severe - MC II and MD.

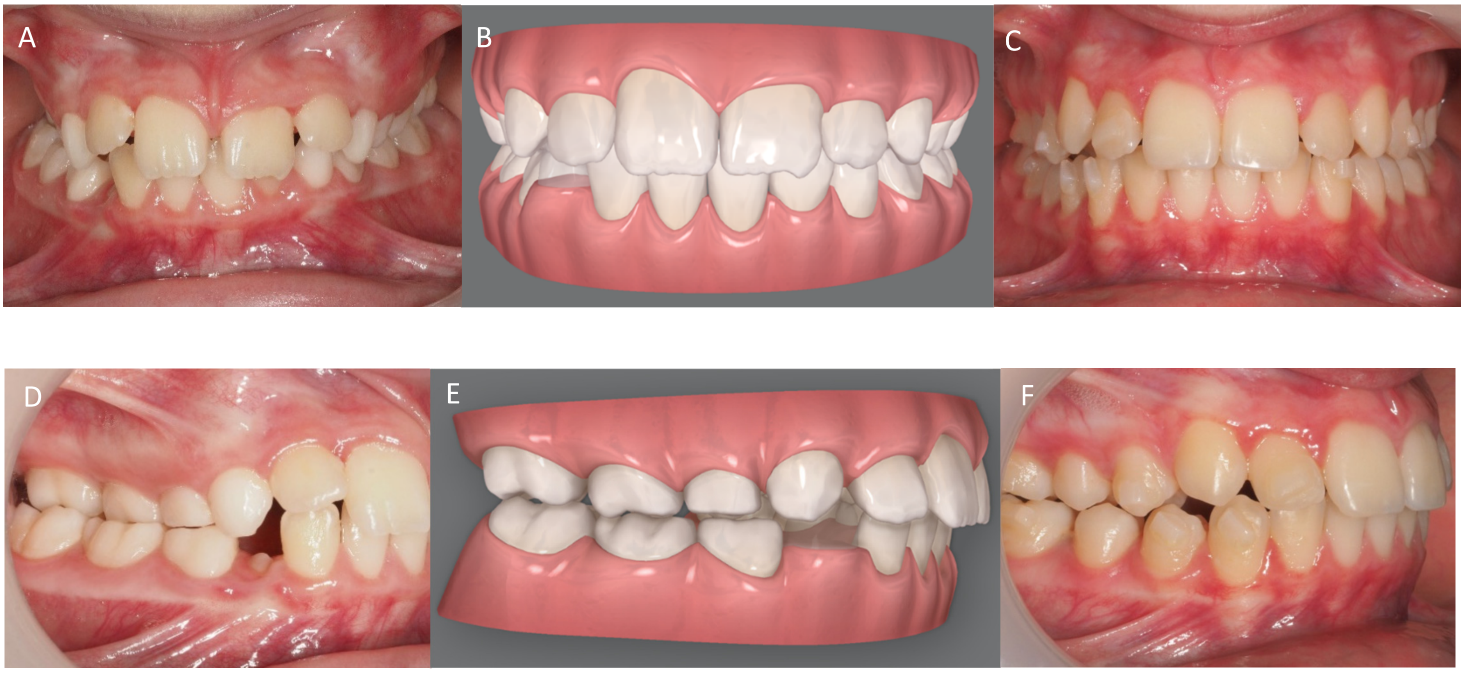

Case 7: Moderate - **MC II**.

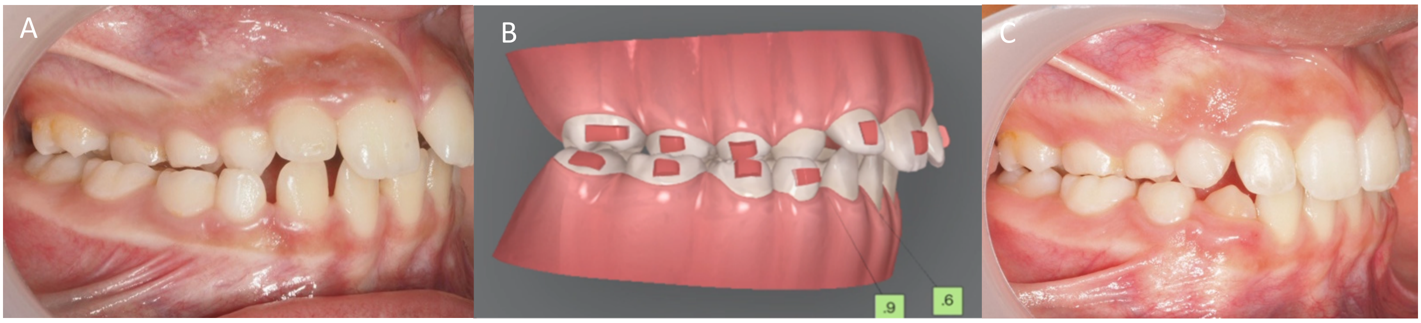

Case 8: Severe – **Cr** and Sagittal skeletal problem.

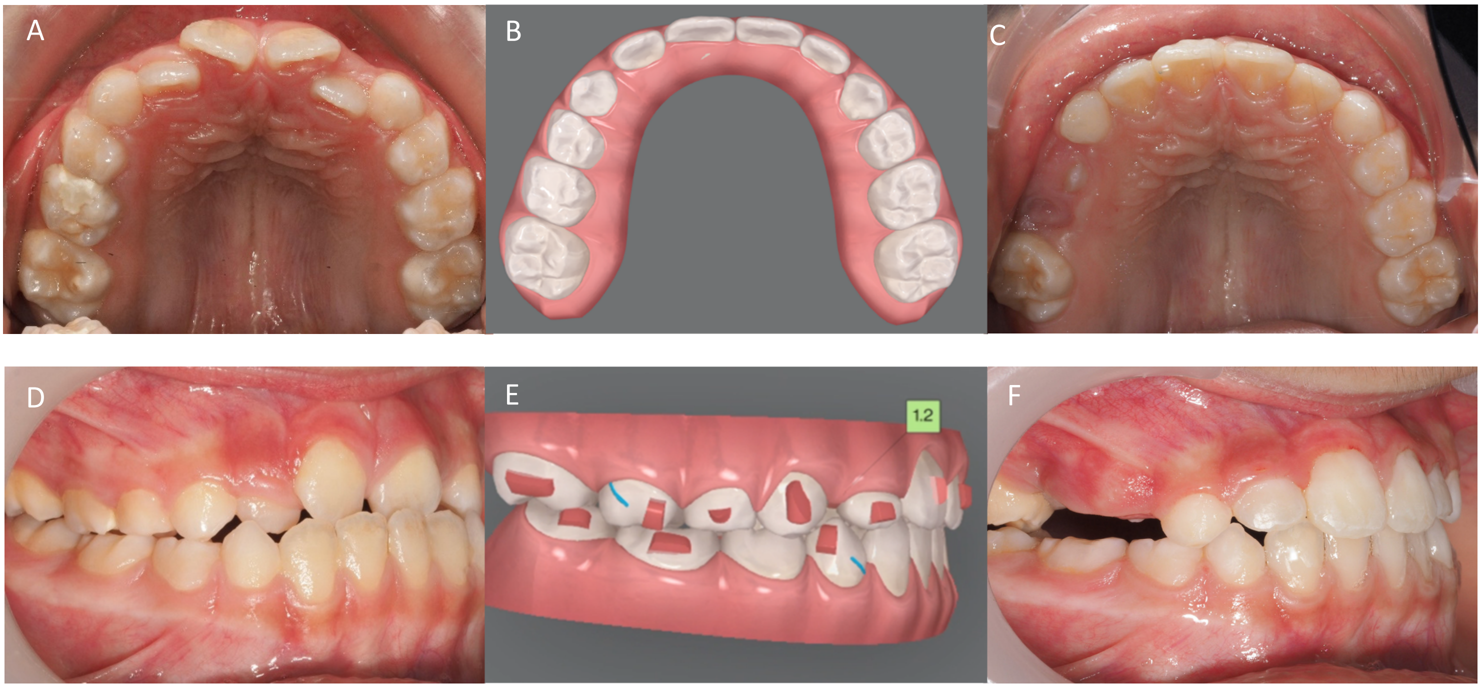

Case 9: Severe - **MD**; Cr.

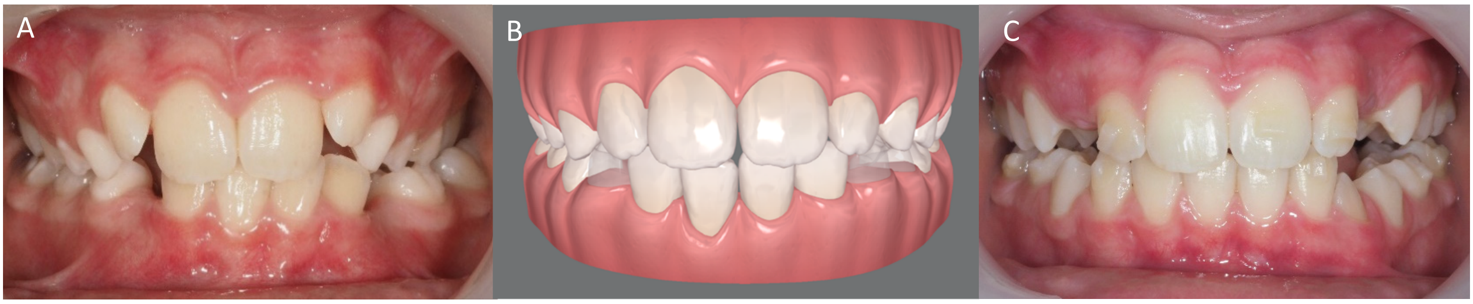

Case 10: Severe - Difficult; DAE; **Cr**; Transversal skeletal problem.

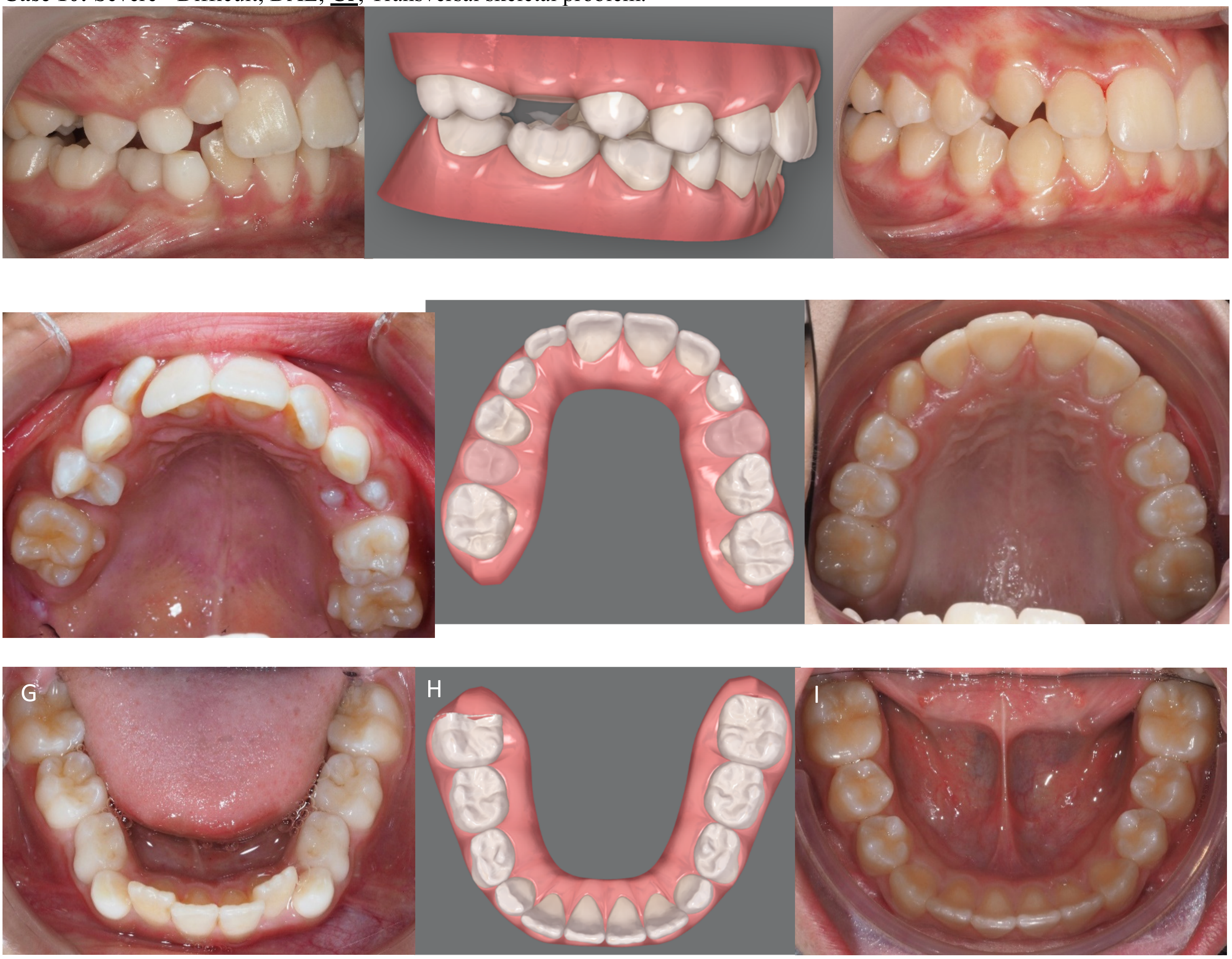

Case 11: Severe - MD and MC II; Sagittal skeletal problem.

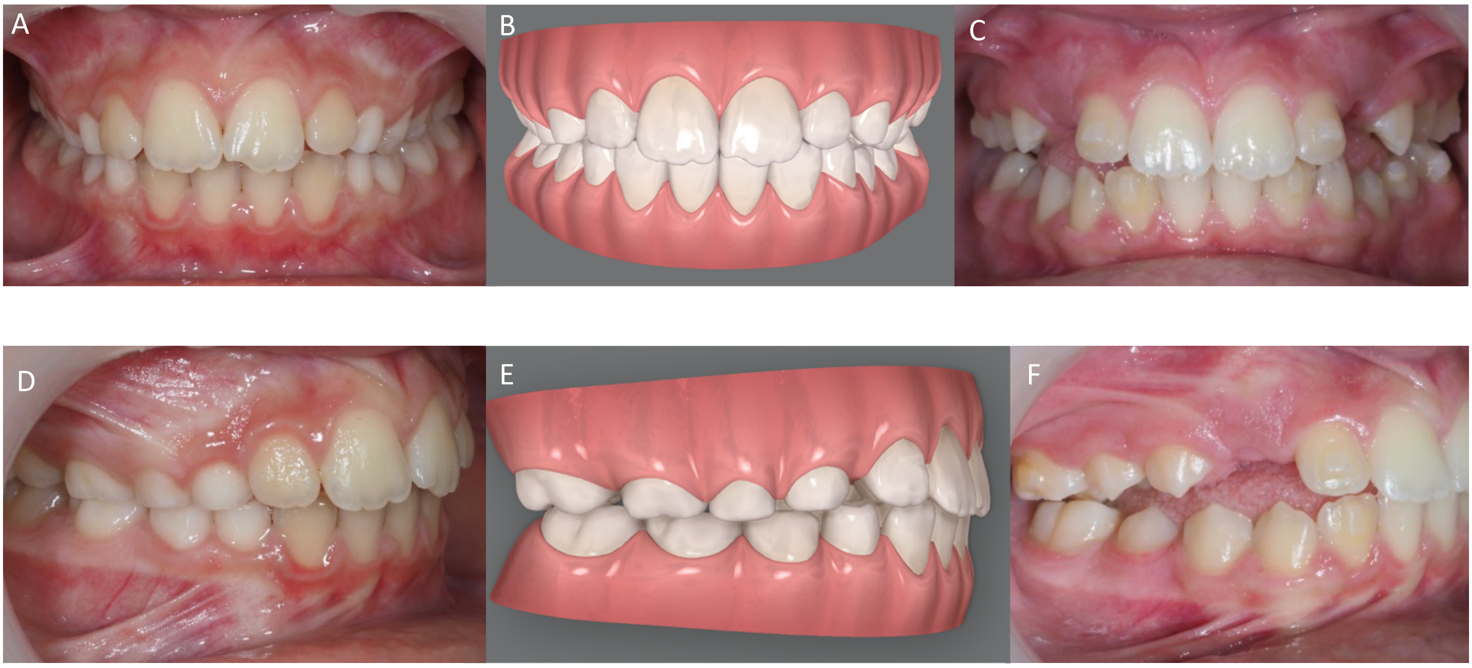

Case 12: Severe - **MDR**; MD; Sagittal and transversal skeletal problem.

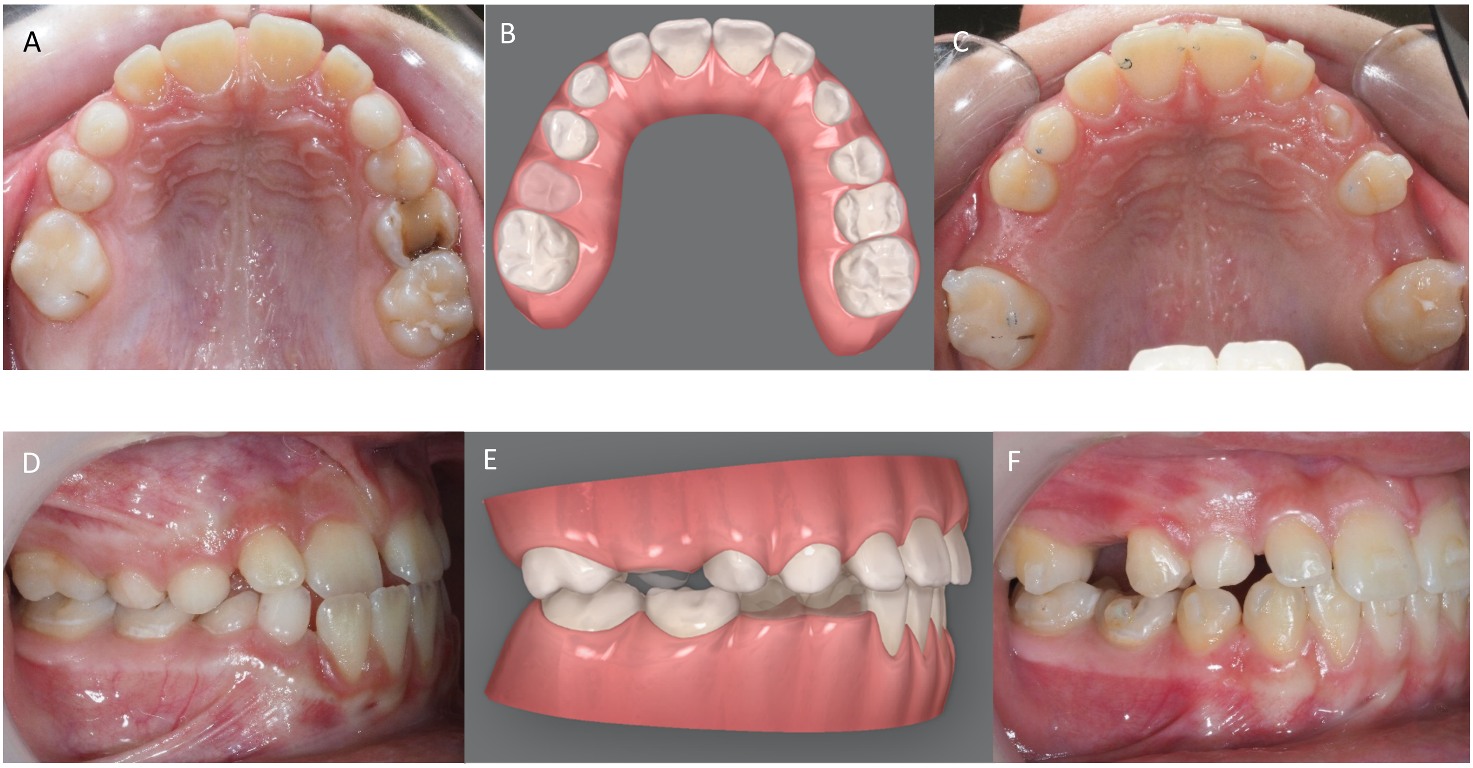

Case 13: Severe – **MD** and Tranversal Skeletal problem

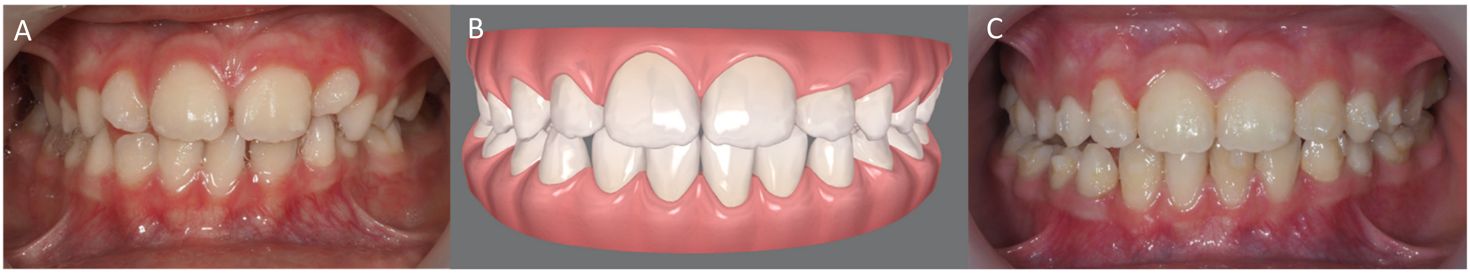

Case 14: Moderate - **MC II**.

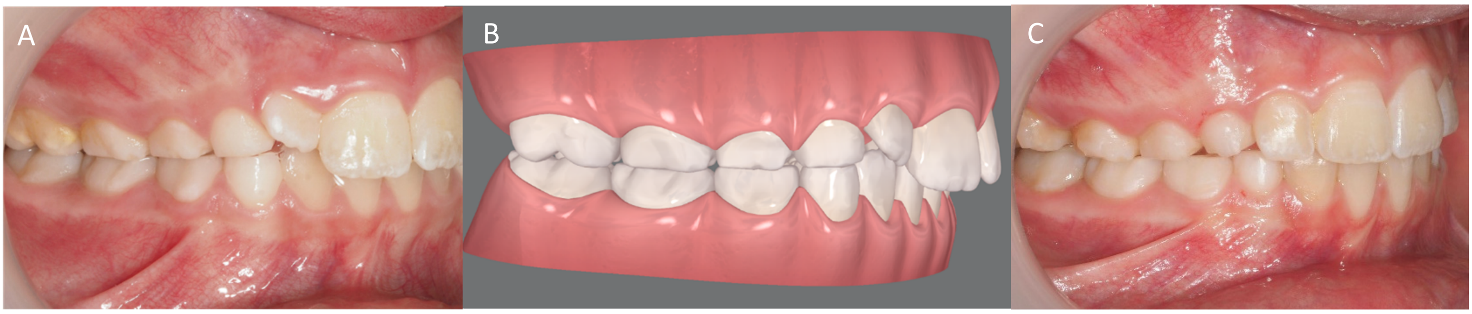

Case 15: Moderate - **PCB**.

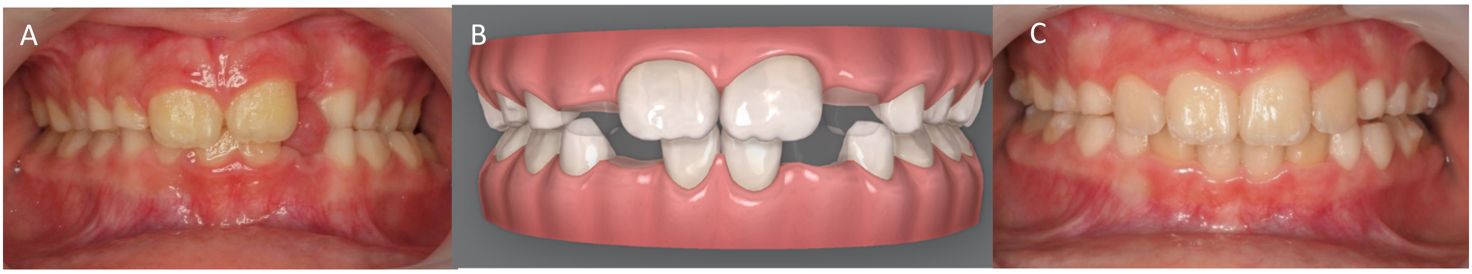

Case 16: Severe - MD; PCB; DAE.

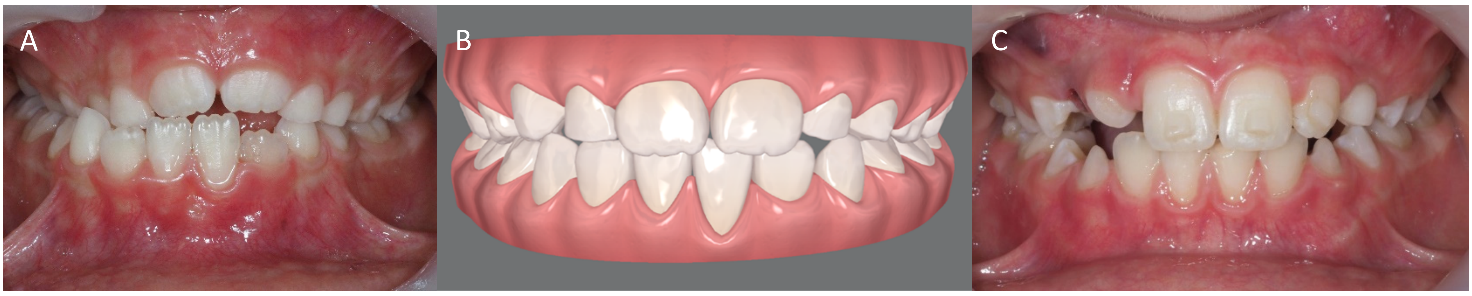

Case 17: Moderate – Cr and Sagittal Skeletal problem.

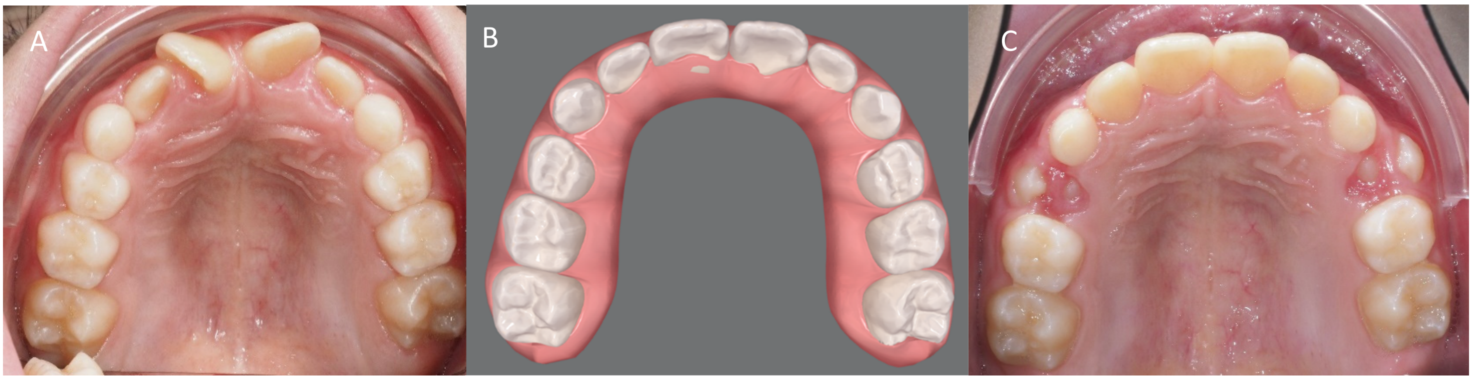

Case 18: Severe - Cr; MD and SR.

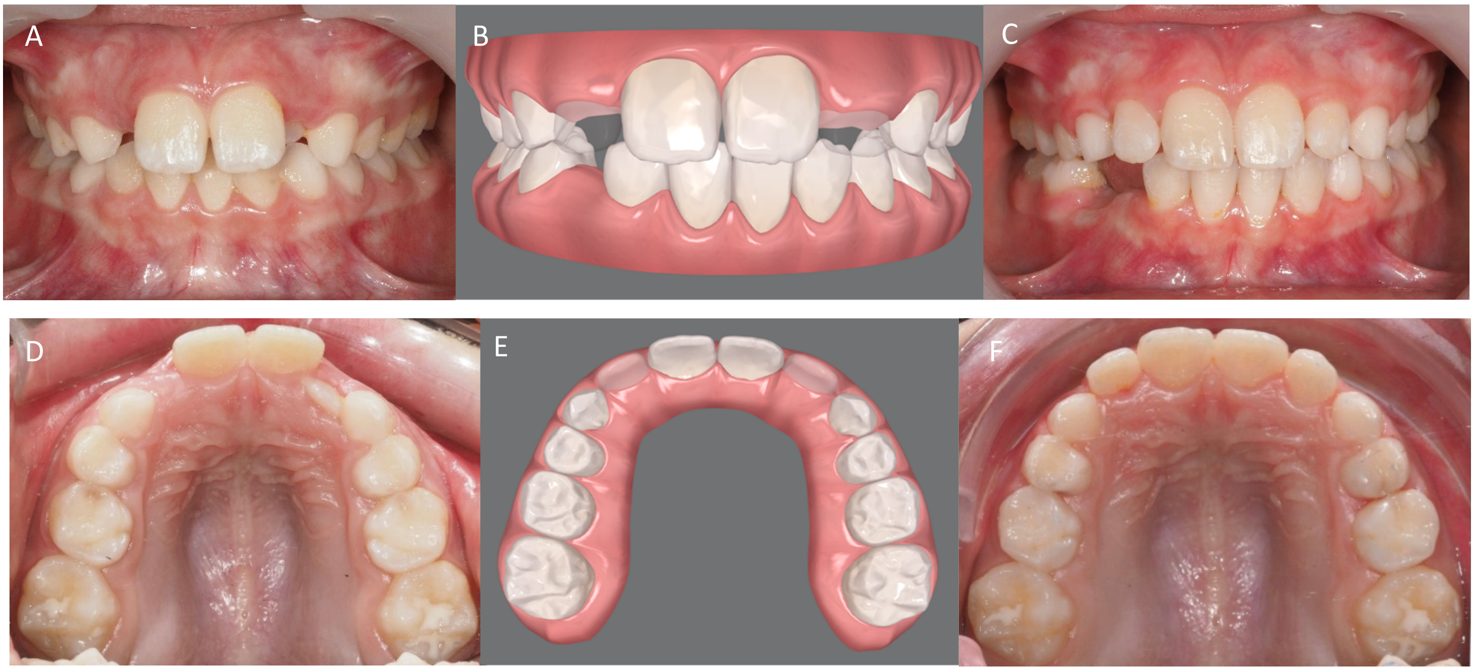

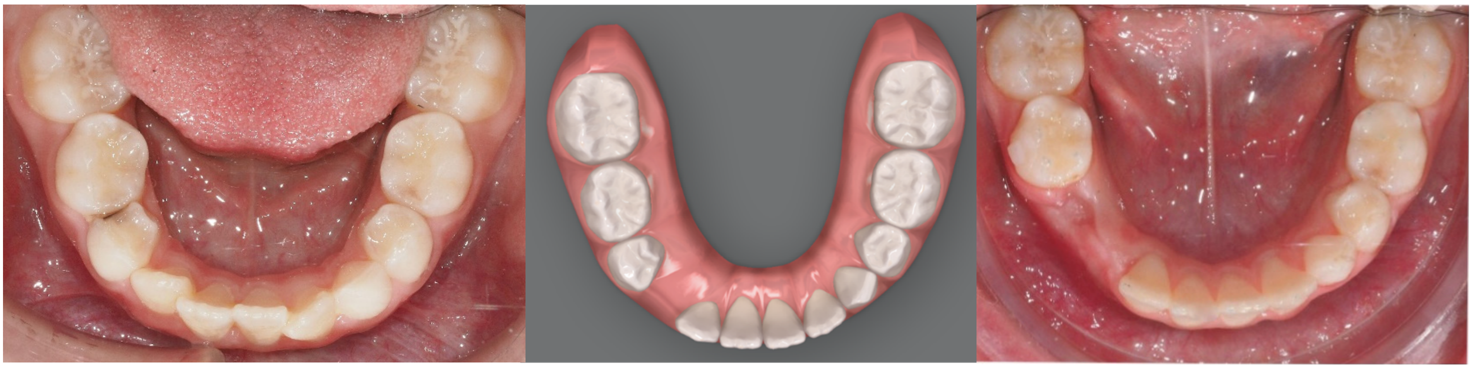

Case 19: Severe - **<u>MD</u>** and Tranversal Skeletal problem.

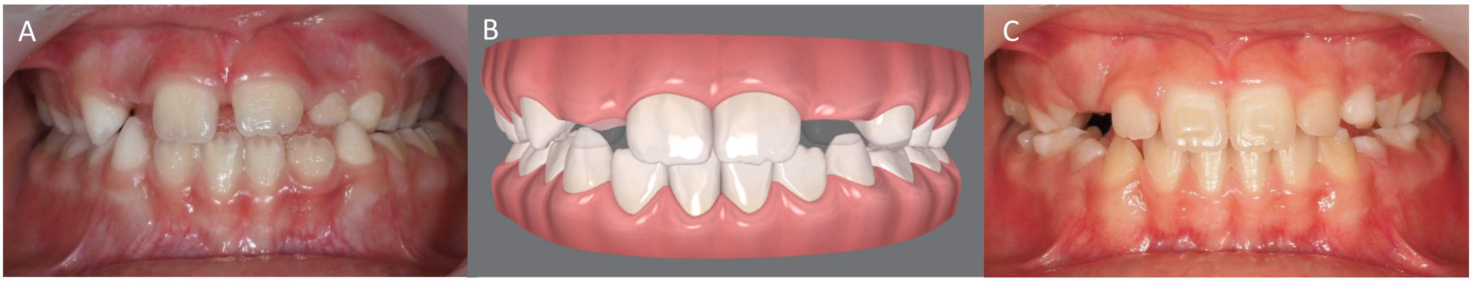

Case 20: Severe - **<u>OB</u>**; Vertical Skeletal problem.

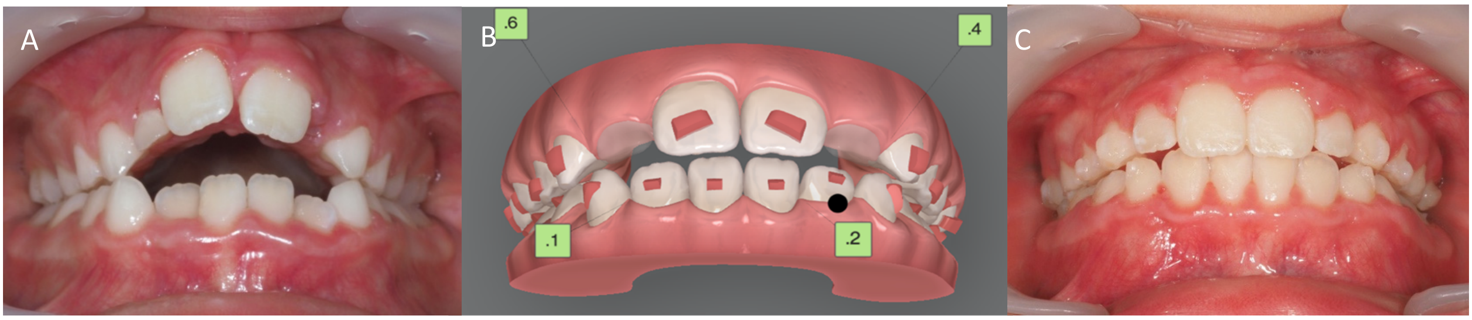

Case 21 (RL): Severe - **<u>MC II</u>**; Sagittal skeletal problem.

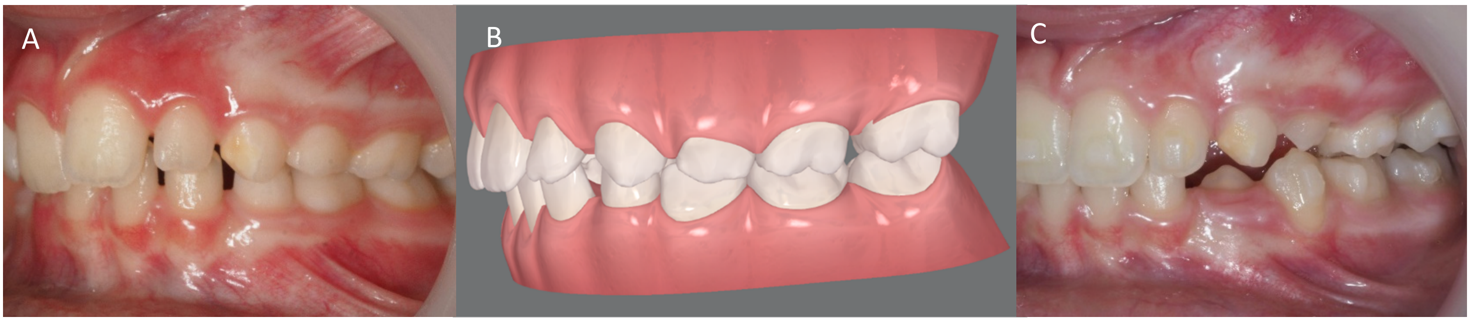

Case 22: Moderate - **<u>PCB</u>**.

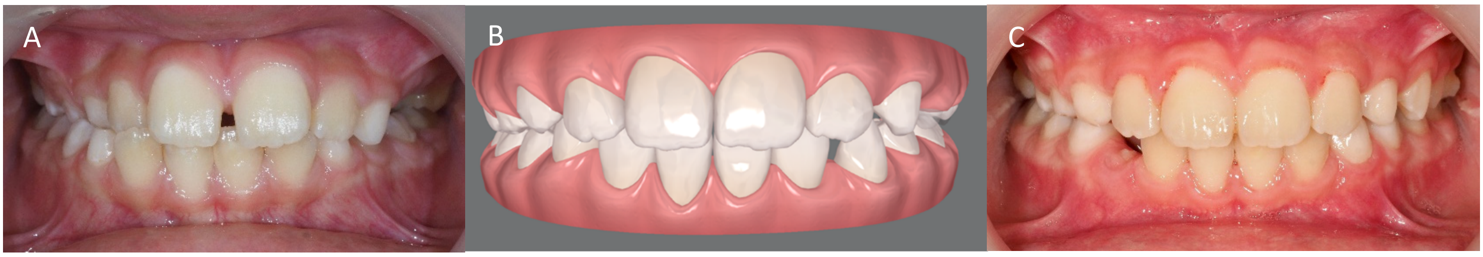

Case 23: Severe - Difficult; **DAE**; SR; MC; MD; Cr; Sagittal and transversal skeletal problem.

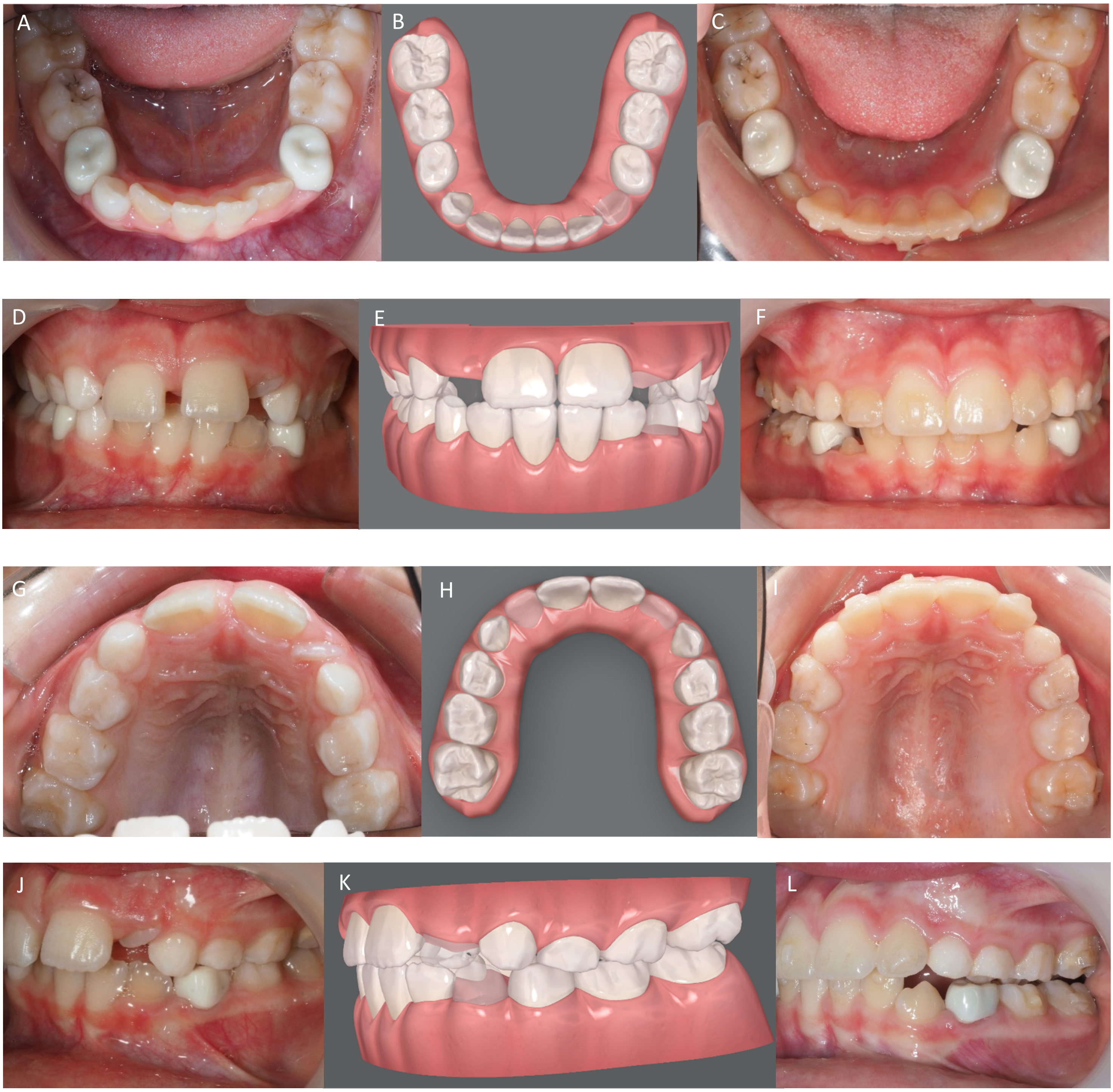

Supplement: Supplementary file 1 [file children-09-01176-s001.zip › Pinho_et_al.-SupplementaryFigureS1_final.pdf]
