# Supplementary material for: Interceptive Treatment with Invisalign® First in Moderate and Severe Cases: A Case Series
Source: Children (Basel). 2022 Aug 5;9(8):1176. doi: 10.3390/children9081176 (PMC9406487; doi:10.3390/children9081176)
Supplement: Supplementary file 1 [file children-09-01176-s001.zip › Pinho_et_al.-SupplementaryFigureS2_final.pdf]

Supplementary Figure S2. Initial (A) and Planned (B) ClinCheck® treatment plans (first set of aligners).

Case 1

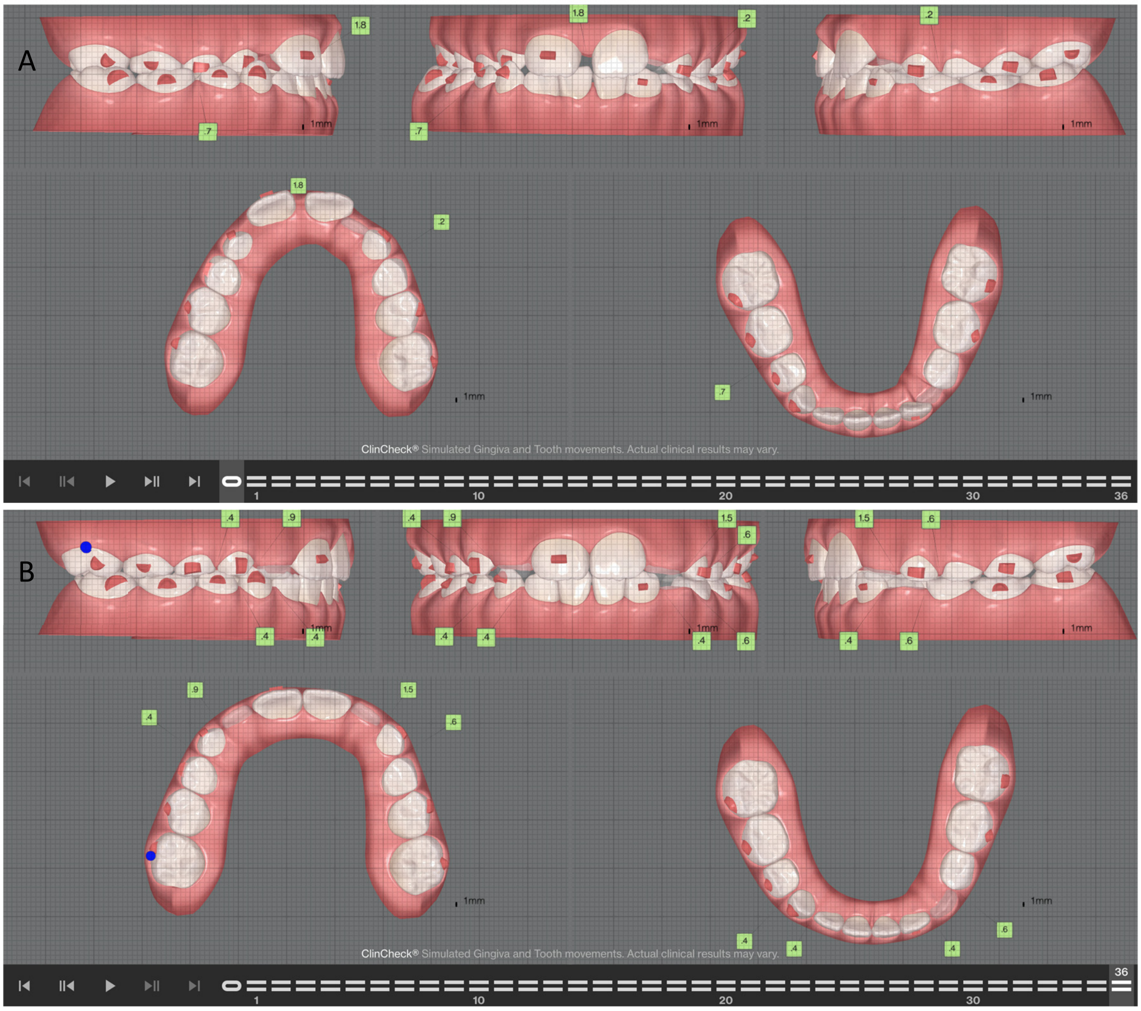

Case 2

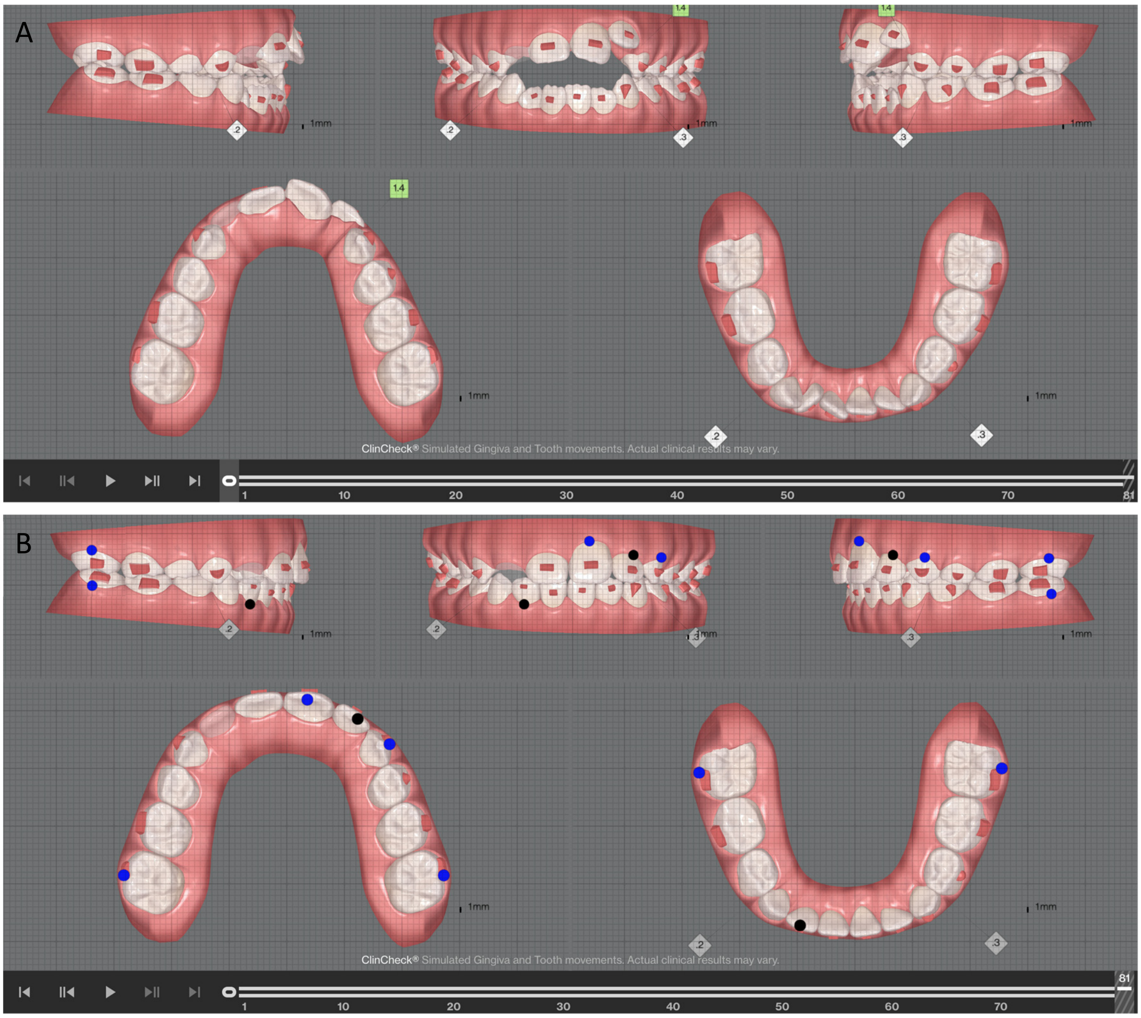

A
1.4
1mm
ClinCheck® Simulated Gingiva and Tooth movements. Actual clinical results may vary.
1
10
20
30
40
50
60
70
81
B
ClinCheck® Simulated Gingiva and Tooth movements. Actual clinical results may vary.

Case 3

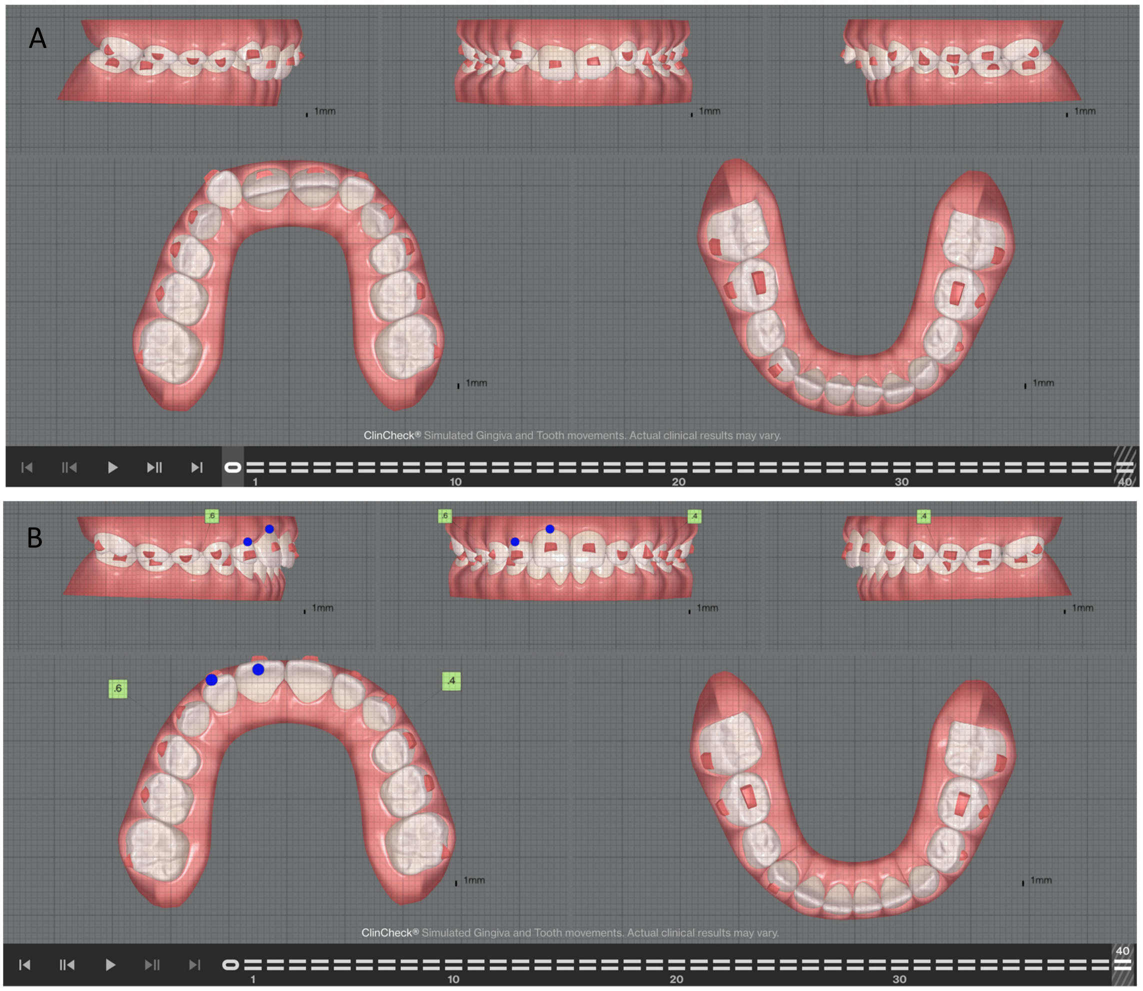
A
1mm
1mm
1mm
1mm
1mm
ClinCheck® Simulated Gingiva and Tooth movements. Actual clinical results may vary.
1
10
20
30
40
B
.6
.6
.4
.4
.6
.4
1mm
1mm
1mm
1mm
1mm
ClinCheck® Simulated Gingiva and Tooth movements. Actual clinical results may vary.
1
10
20
30
40

Case 4

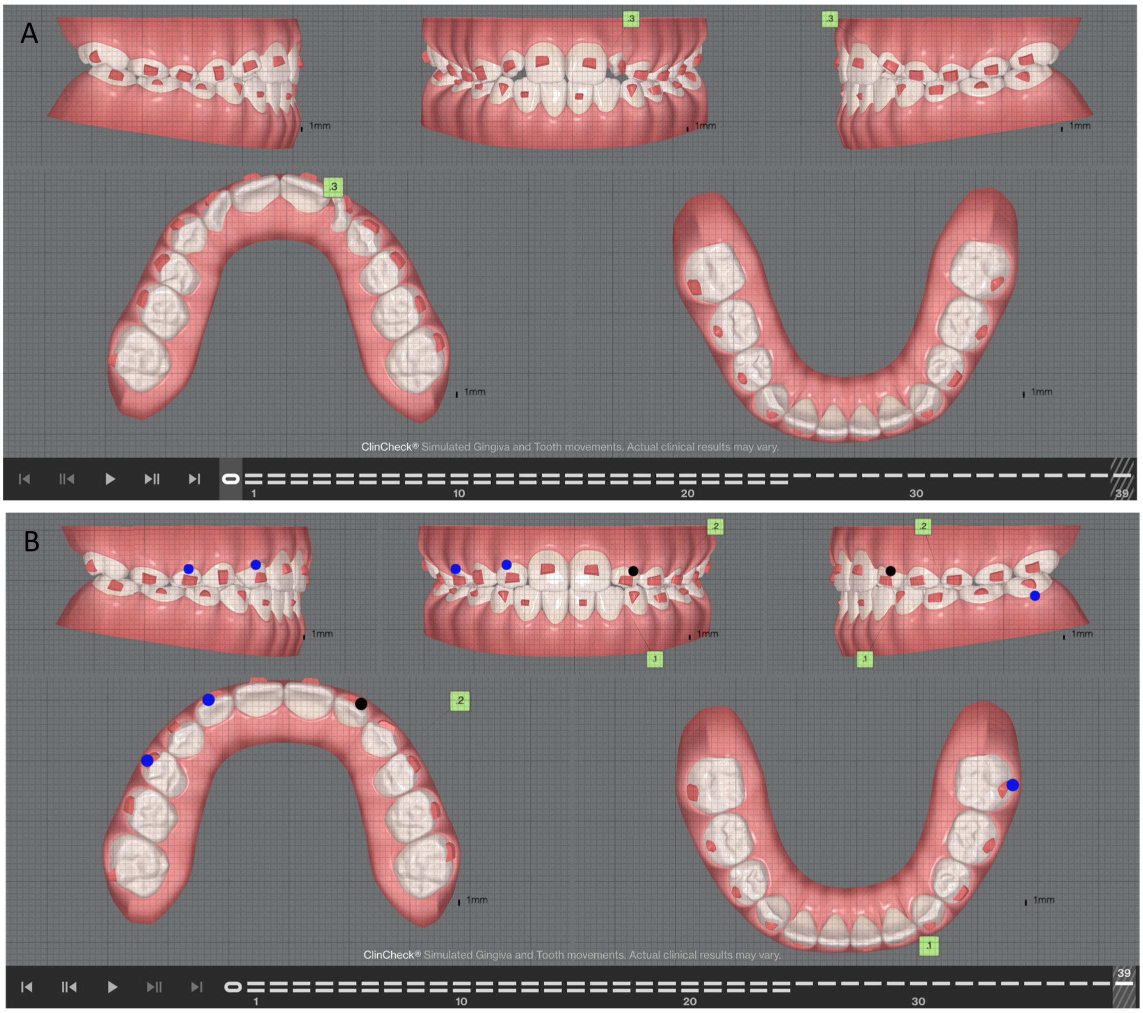

A
B
ClinCheck® Simulated Gingiva and Tooth movements. Actual clinical results may vary.
1mm
1
10
20
30
39

Case 5

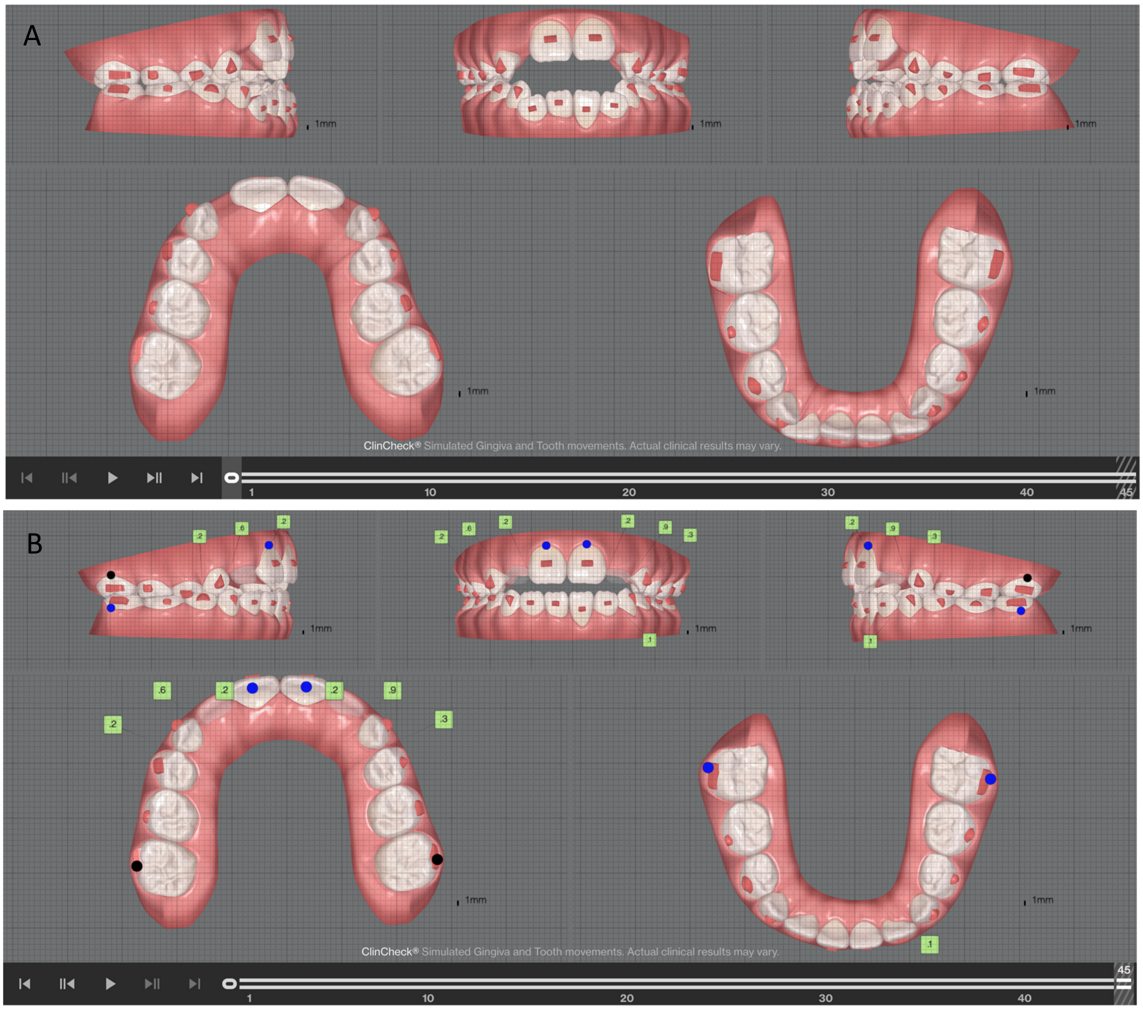
A
B
ClinCheck® Simulated Gingiva and Tooth movements. Actual clinical results may vary.
1mm

Case 6

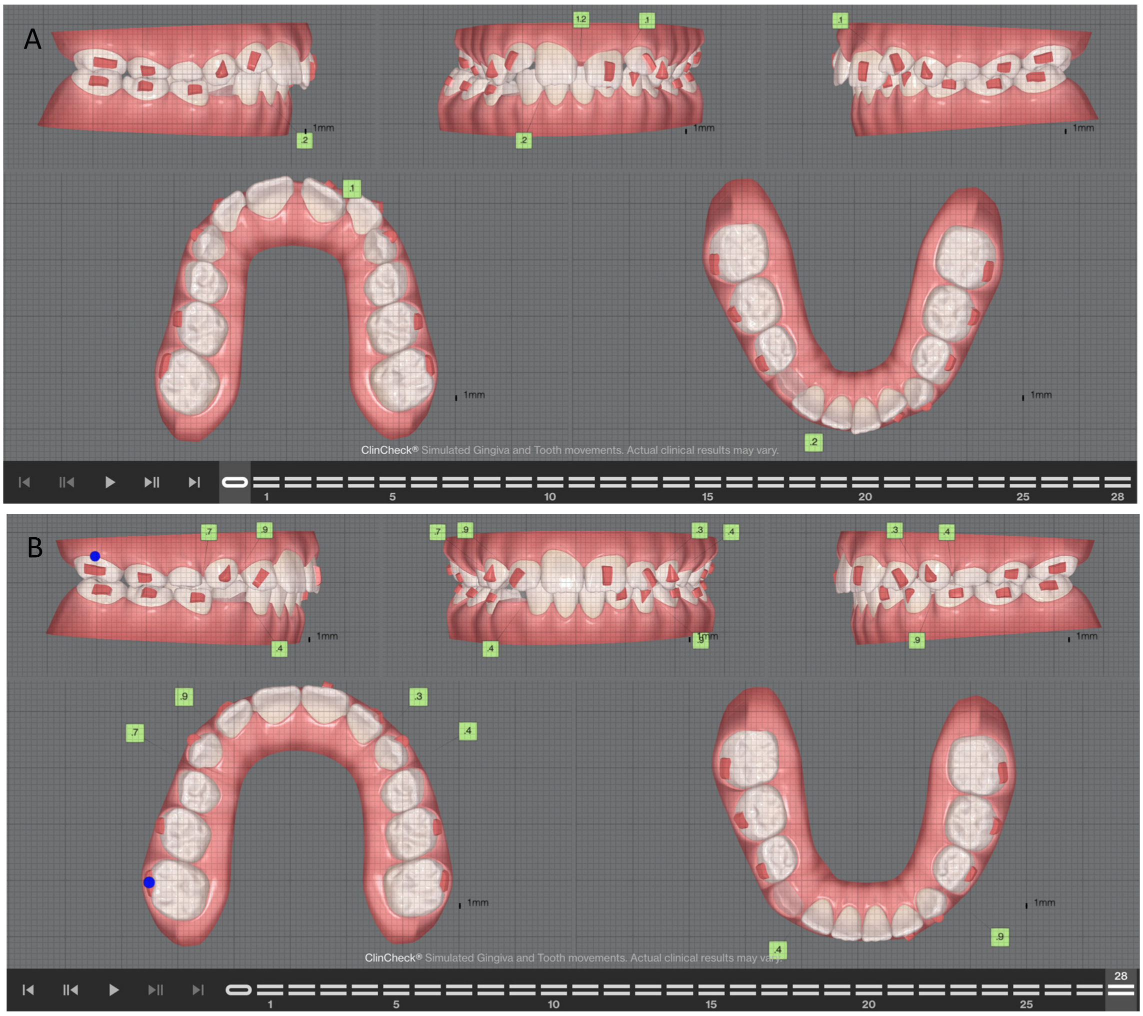
A
ClinCheck® Simulated Gingiva and Tooth movements. Actual clinical results may vary.
B
ClinCheck® Simulated Gingiva and Tooth movements. Actual clinical results may vary.

Case 7

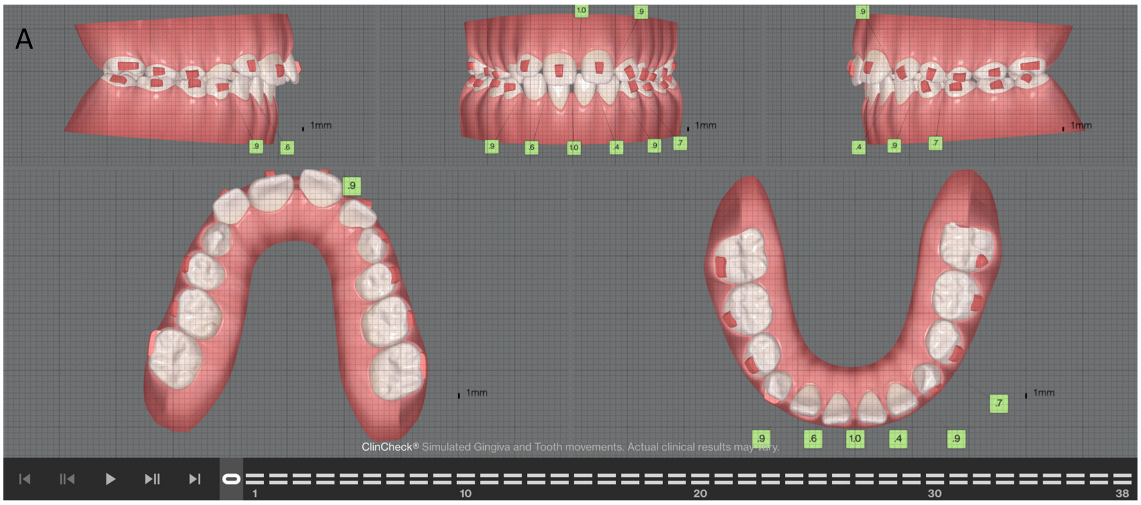
A
ClinCheck® Simulated Gingiva and Tooth movements. Actual clinical results may vary.

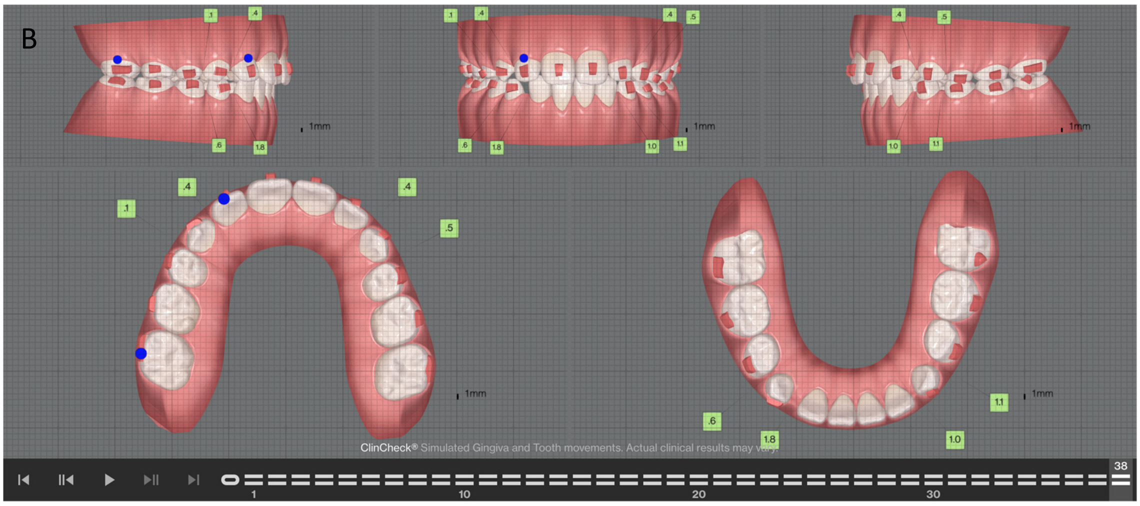
B
ClinCheck® Simulated Gingiva and Tooth movements. Actual clinical results may vary.

Case 8

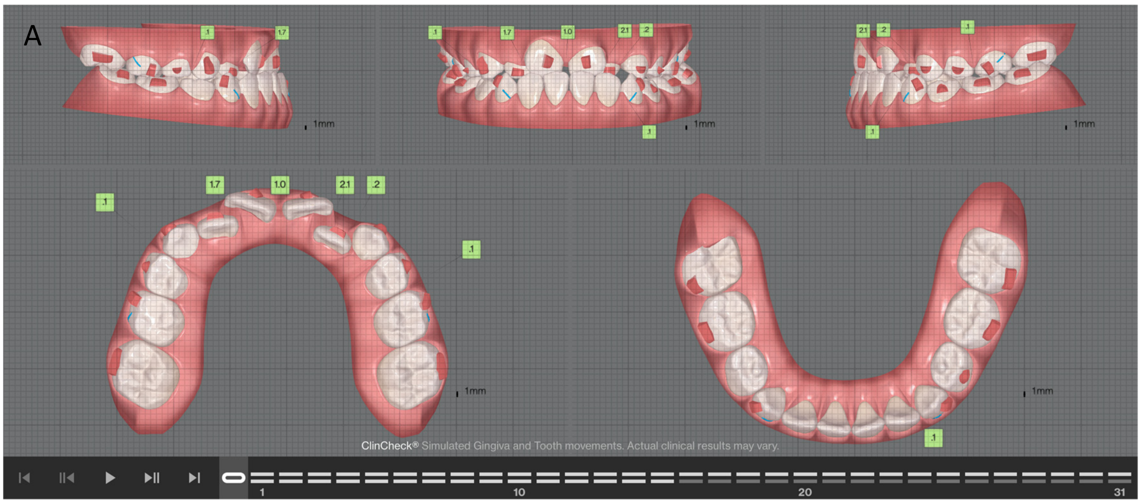
A
ClinCheck® Simulated Gingiva and Tooth movements. Actual clinical results may vary.

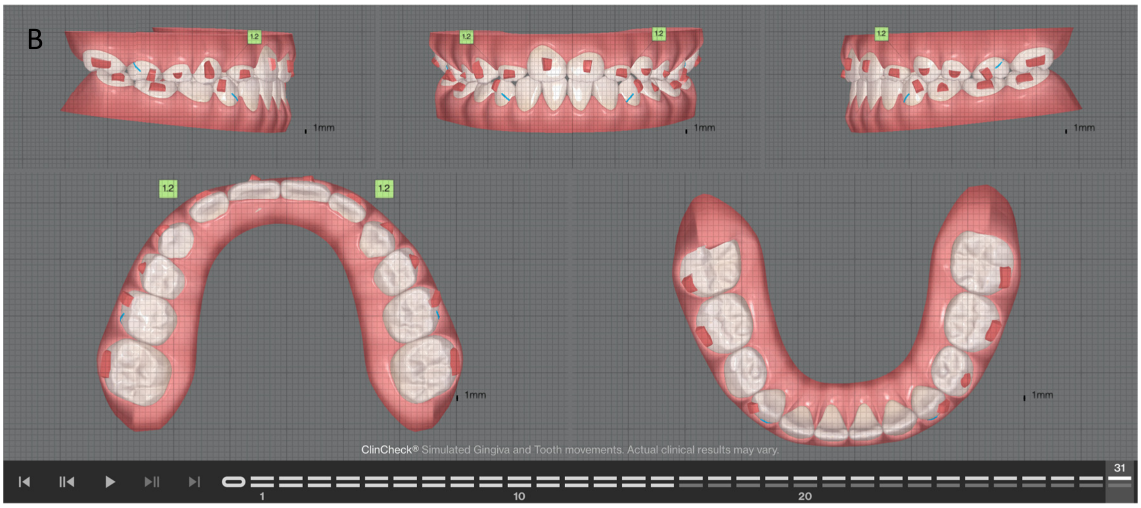
B
ClinCheck® Simulated Gingiva and Tooth movements. Actual clinical results may vary.

Case 9

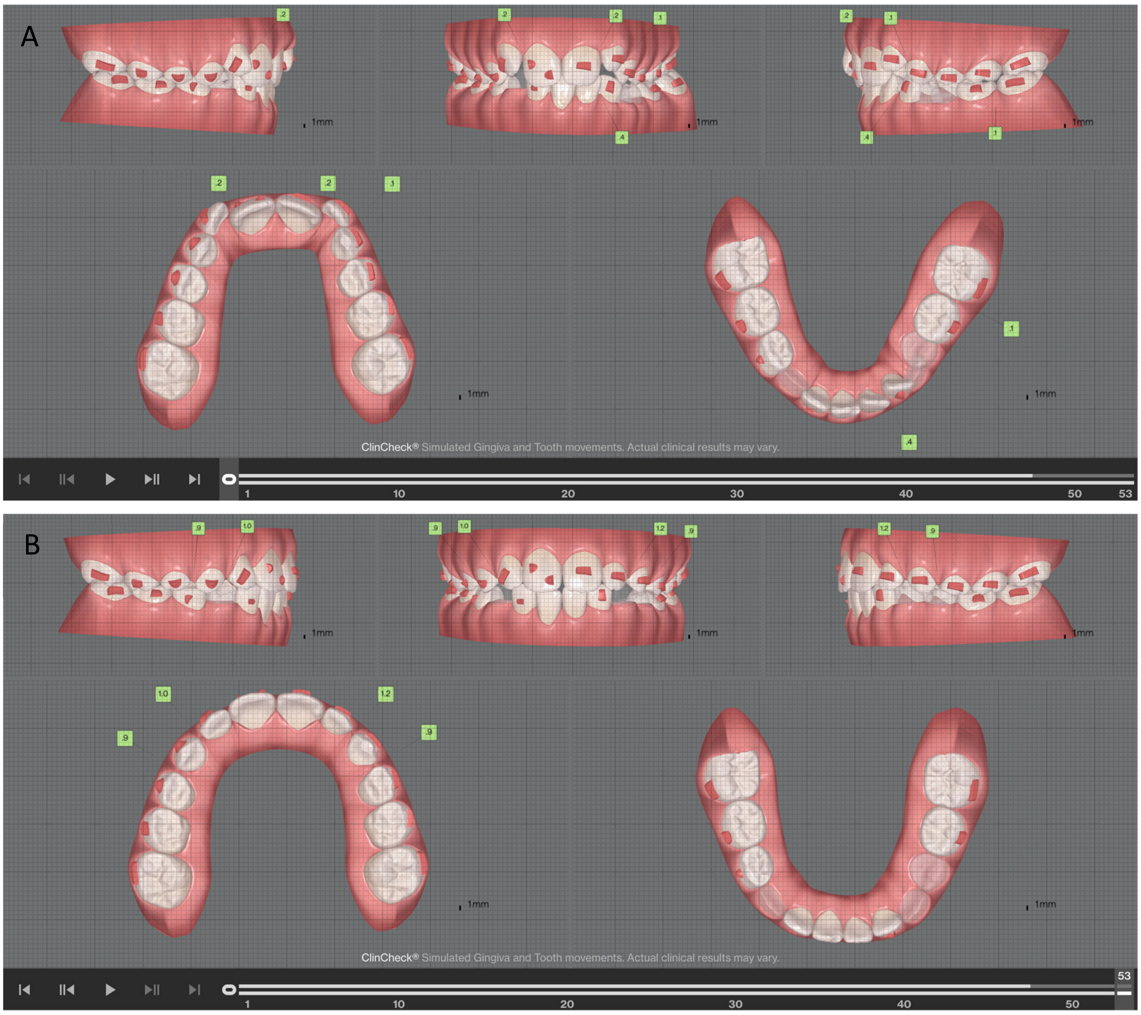
A
1mm
ClinCheck® Simulated Gingiva and Tooth movements. Actual clinical results may vary.
B
1mm
ClinCheck® Simulated Gingiva and Tooth movements. Actual clinical results may vary.

Case 10

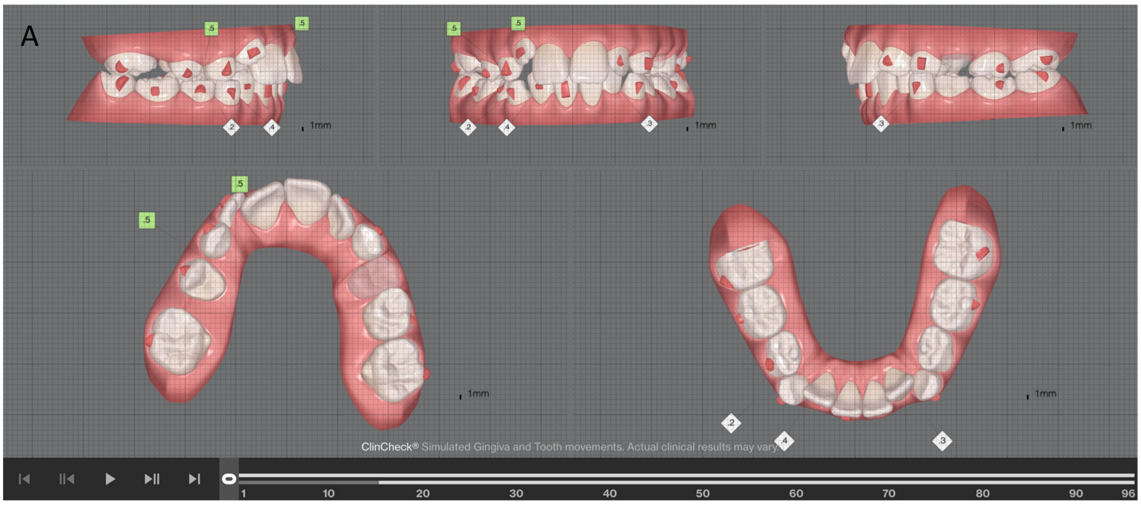
A
1mm
ClinCheck® Simulated Gingiva and Tooth movements. Actual clinical results may vary.
1
10
20
30
40
50
60
70
80
90
96

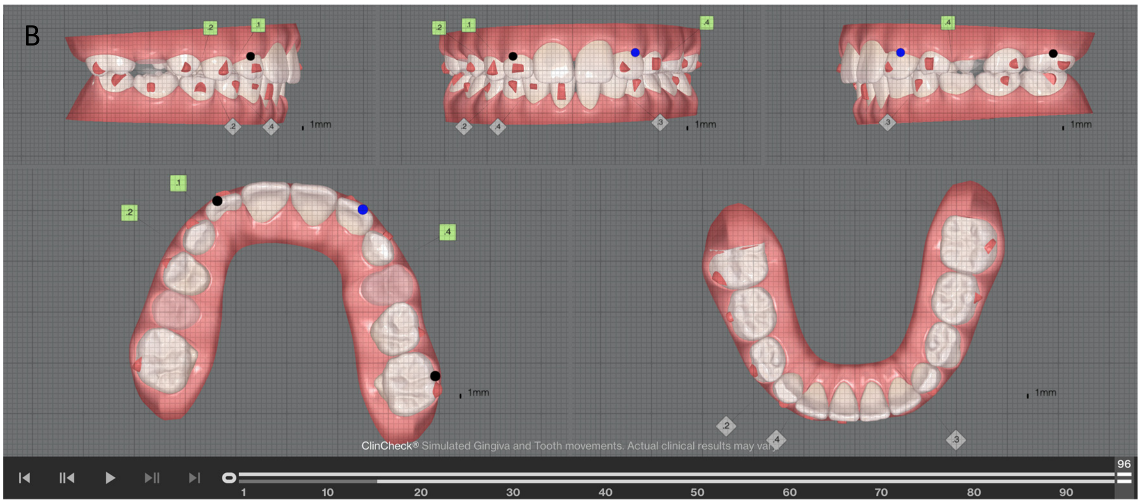
B
1mm
ClinCheck® Simulated Gingiva and Tooth movements. Actual clinical results may vary.
96
1
10
20
30
40
50
60
70
80
90

Case 11

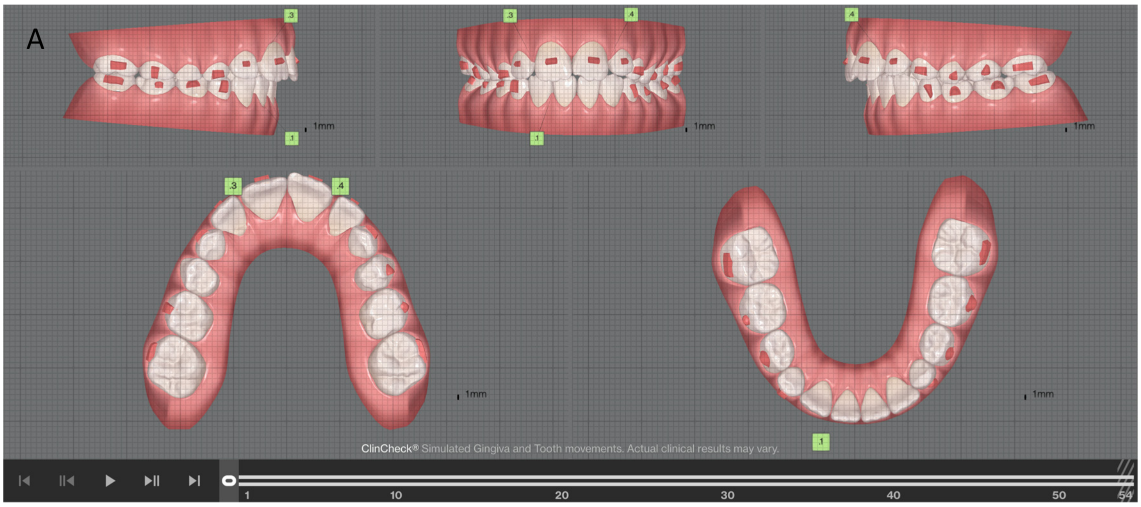
A
ClinCheck® Simulated Gingiva and Tooth movements. Actual clinical results may vary.

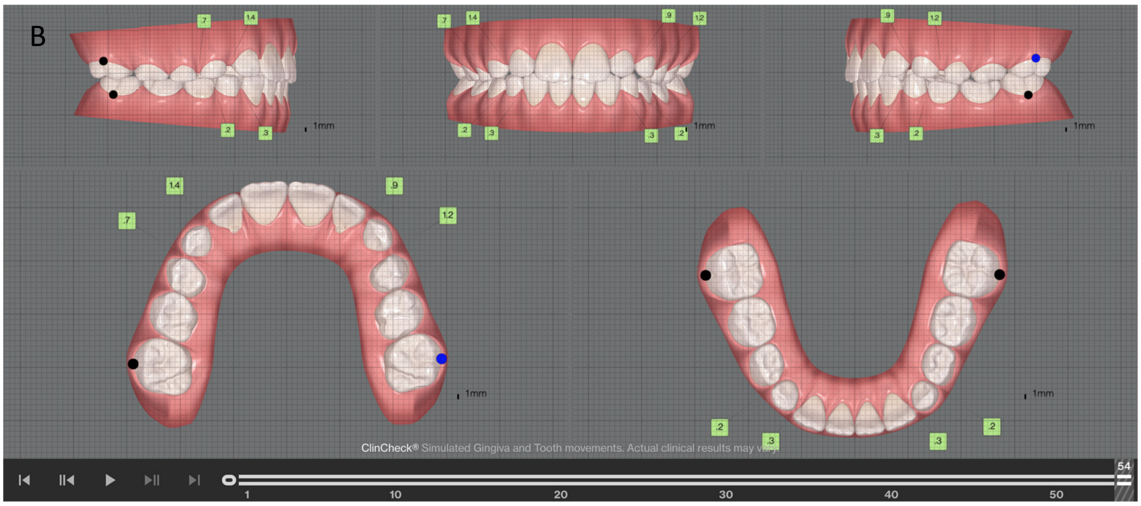
B
ClinCheck® Simulated Gingiva and Tooth movements. Actual clinical results may vary.

Case 12

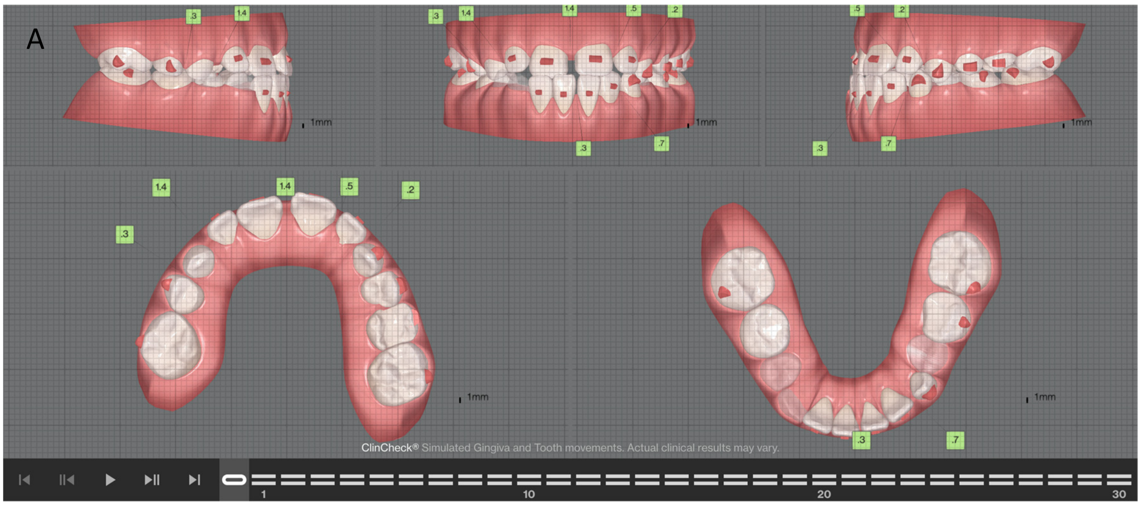
A
ClinCheck® Simulated Gingiva and Tooth movements. Actual clinical results may vary.

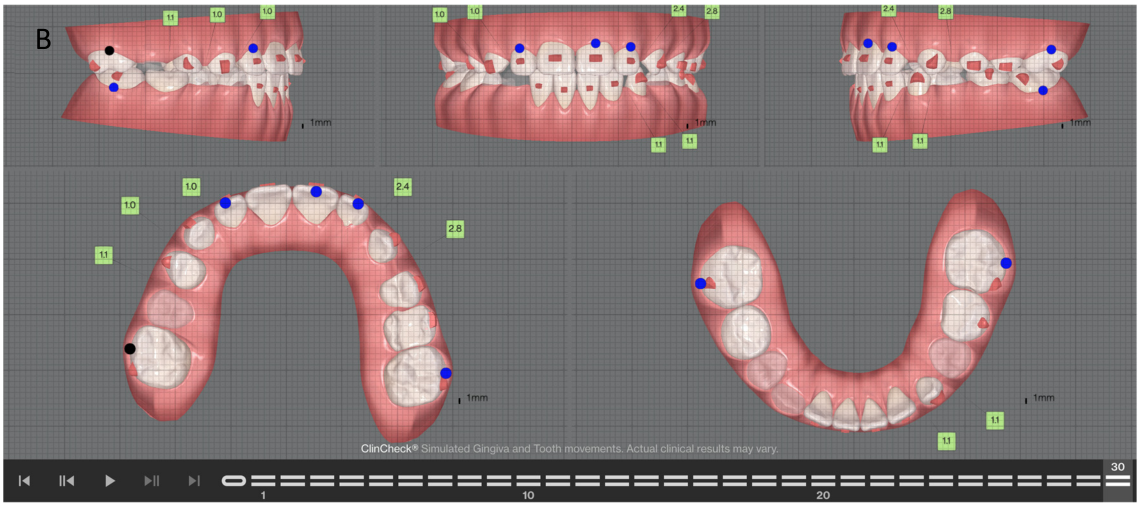
B
ClinCheck® Simulated Gingiva and Tooth movements. Actual clinical results may vary.

Case 13

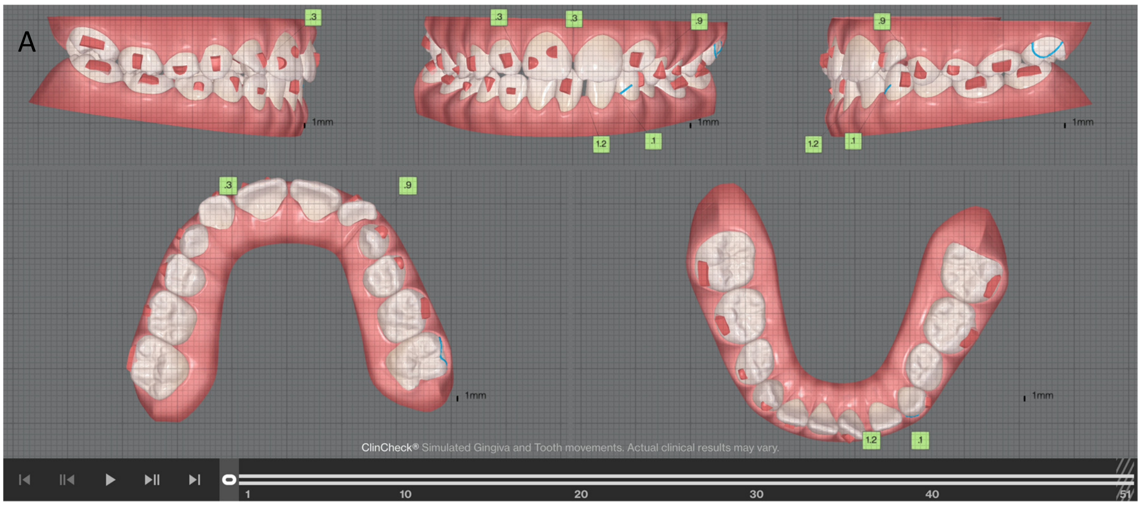
A
ClinCheck® Simulated Gingiva and Tooth movements. Actual clinical results may vary.

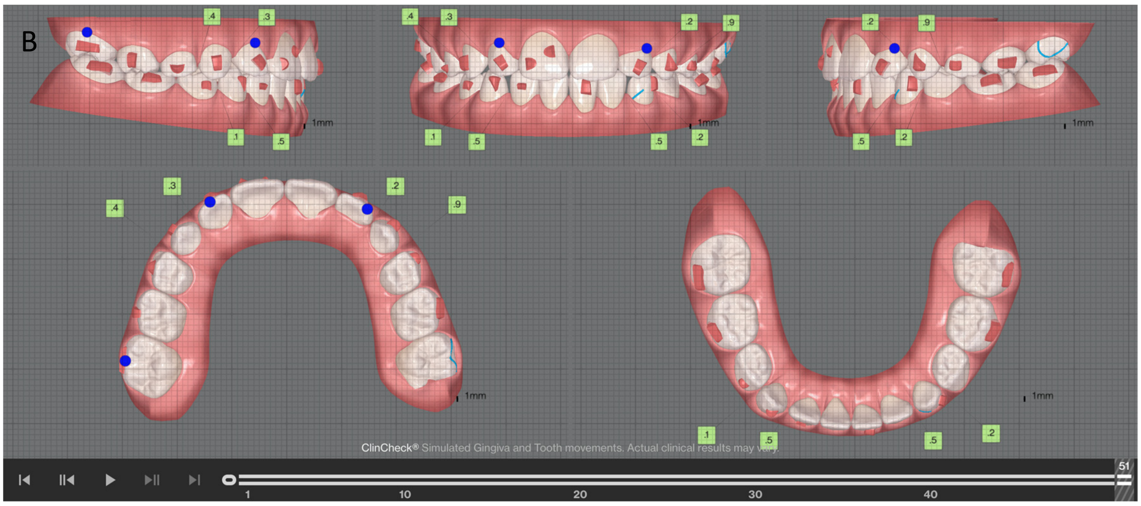
B
ClinCheck® Simulated Gingiva and Tooth movements. Actual clinical results may vary.

Case 14

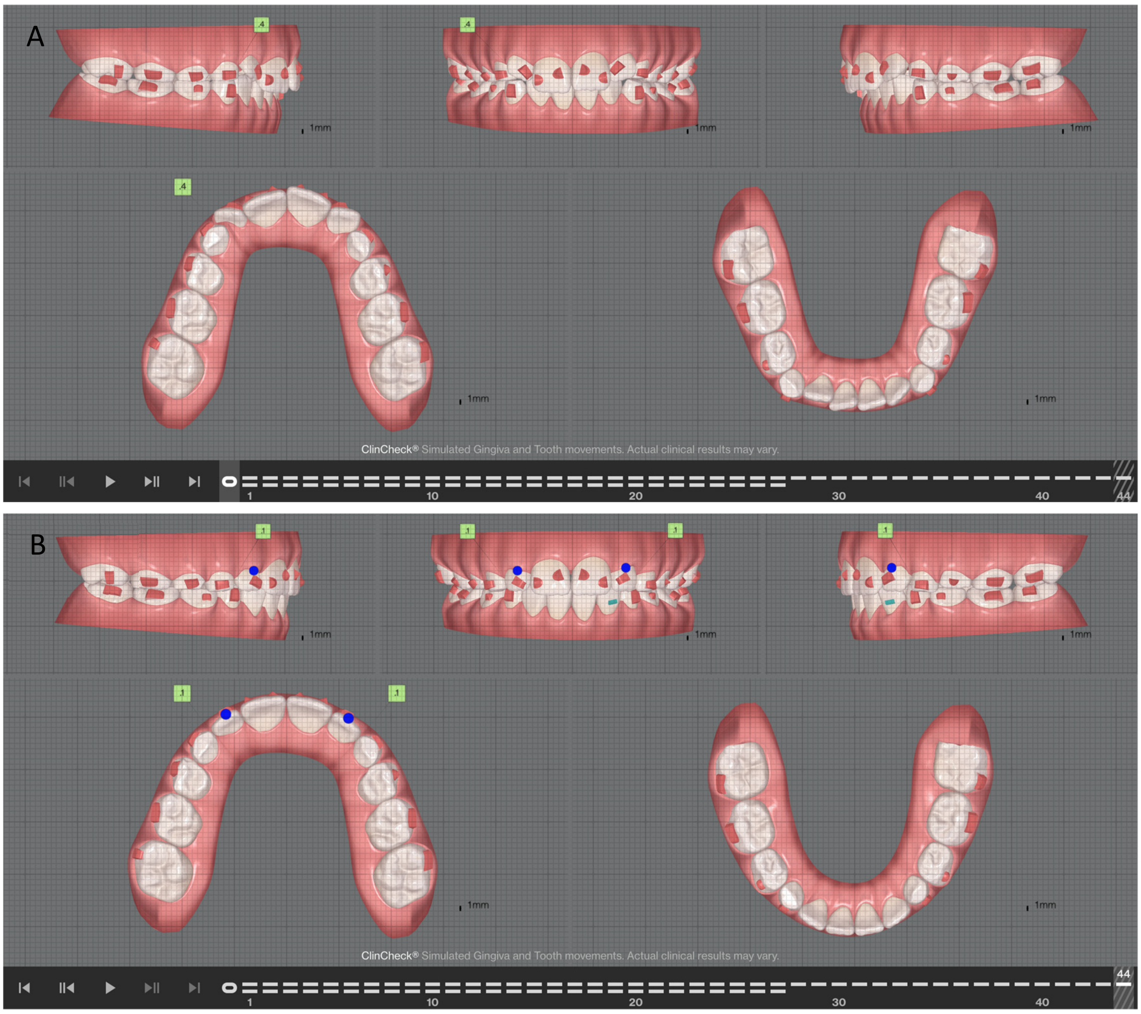
A
B
ClinCheck® Simulated Gingiva and Tooth movements. Actual clinical results may vary.
1mm

Case 15

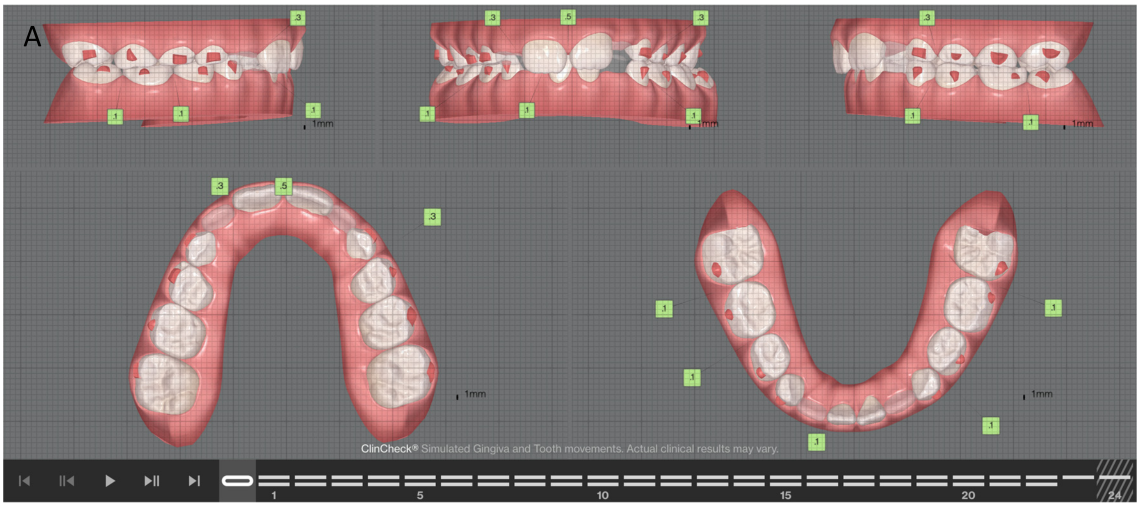
A
ClinCheck® Simulated Gingiva and Tooth movements. Actual clinical results may vary.

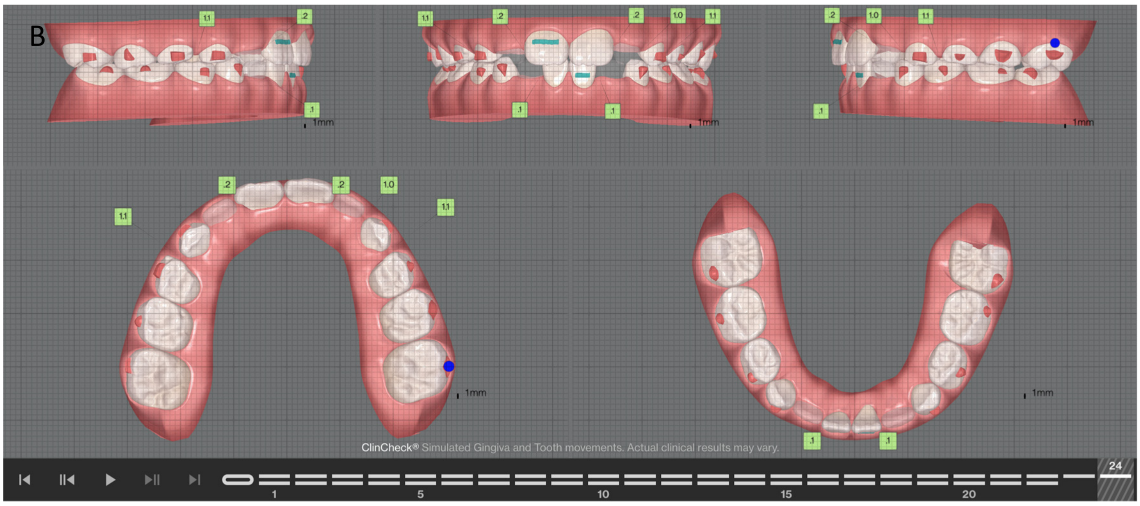
B
ClinCheck® Simulated Gingiva and Tooth movements. Actual clinical results may vary.

Case 16

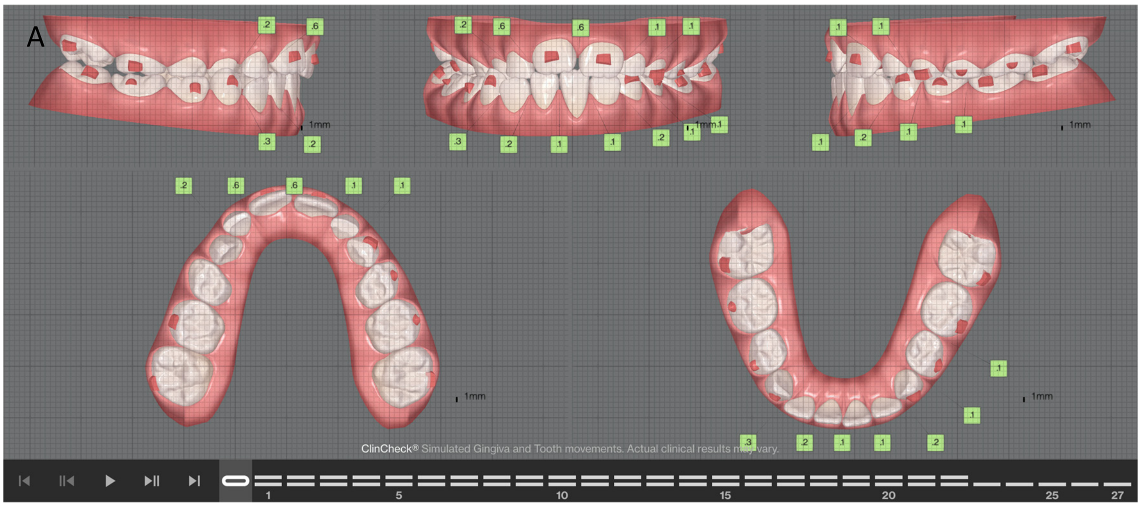
A
ClinCheck® Simulated Gingiva and Tooth movements. Actual clinical results may vary.

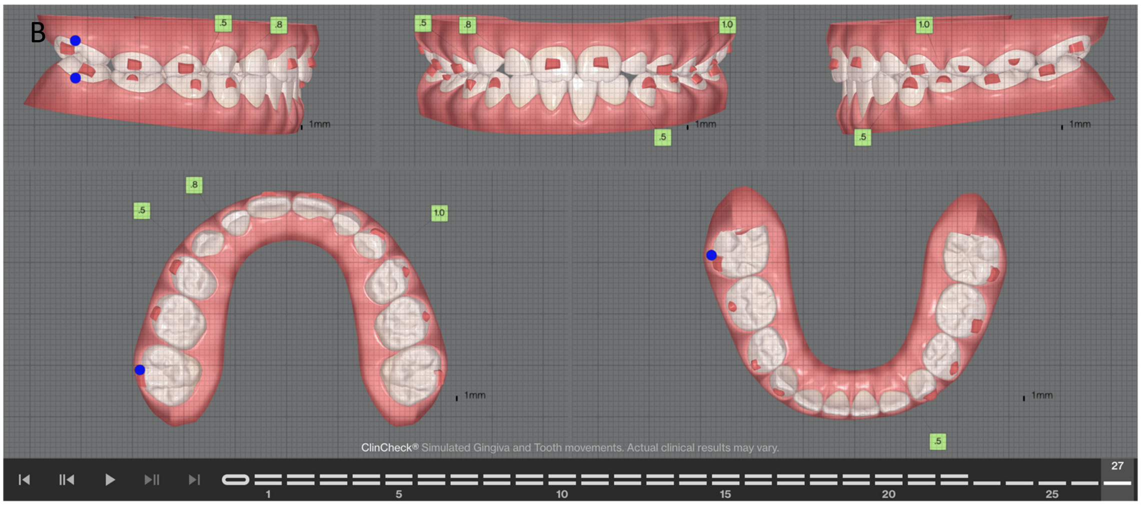
B
ClinCheck® Simulated Gingiva and Tooth movements. Actual clinical results may vary.

Case 17

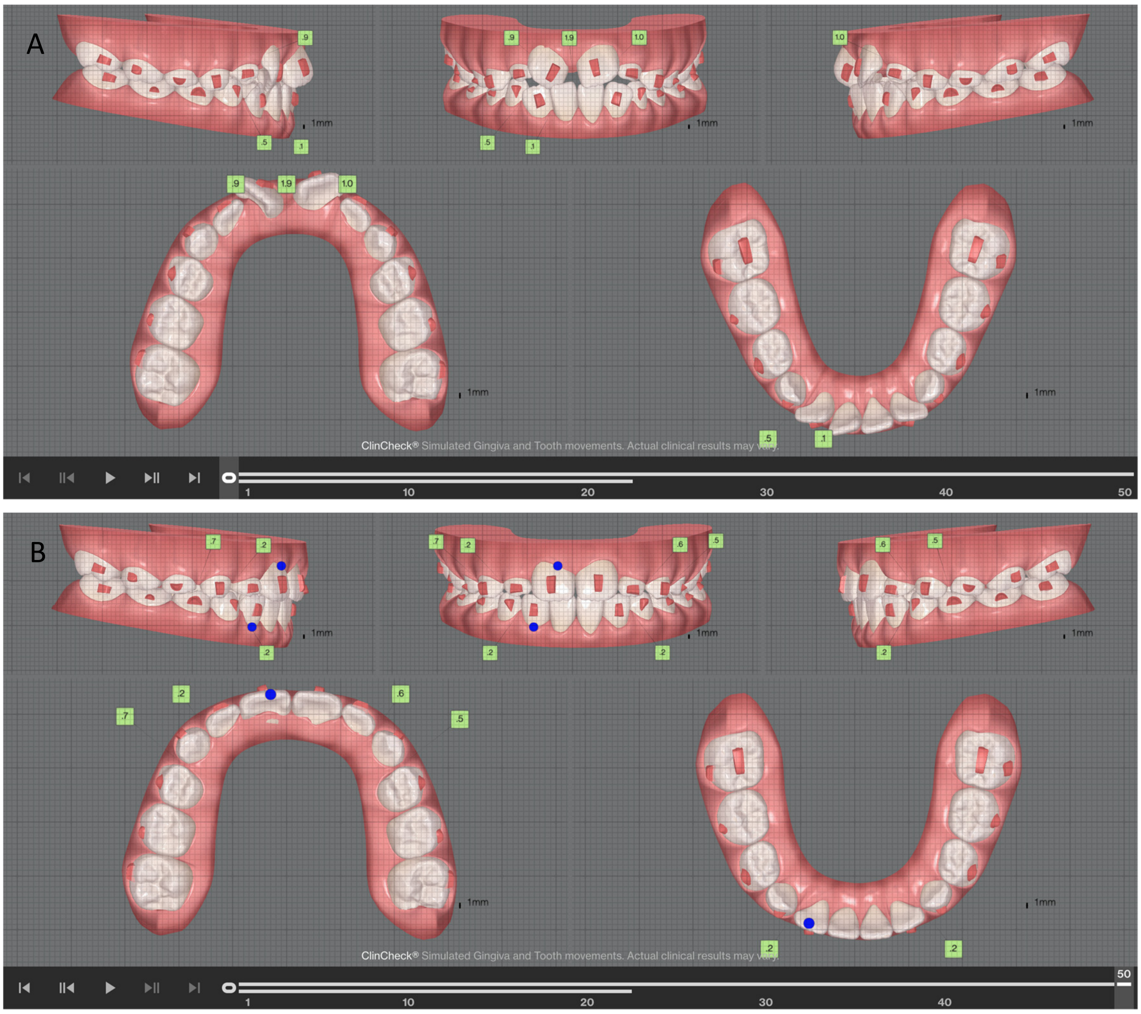
A
B
ClinCheck® Simulated Gingiva and Tooth movements. Actual clinical results may vary.
1mm

Case 18

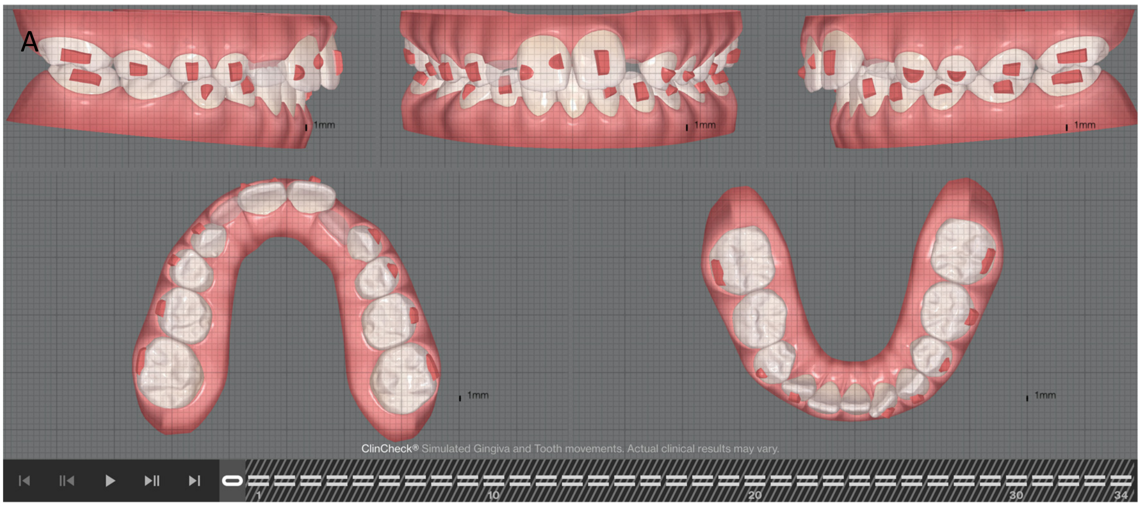
A
1mm
1mm
1mm
1mm
1mm
ClinCheck® Simulated Gingiva and Tooth movements. Actual clinical results may vary.
0
1
10
20
30
34

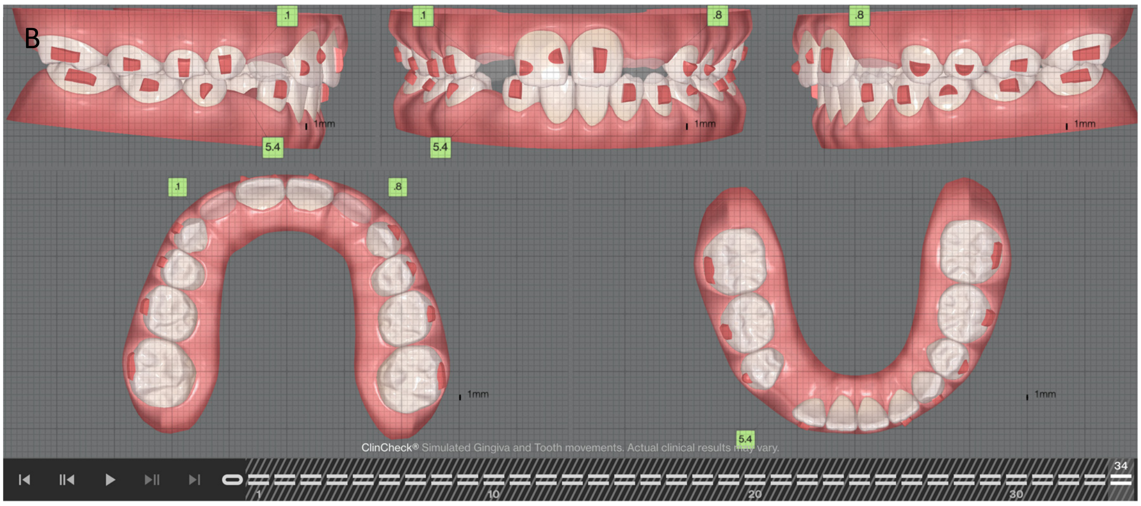
B
.1
.1
.8
.8
5.4
5.4
.1
.8
1mm
1mm
1mm
1mm
1mm
5.4
ClinCheck® Simulated Gingiva and Tooth movements. Actual clinical results may vary.
0
1
10
20
30
34

Case 19

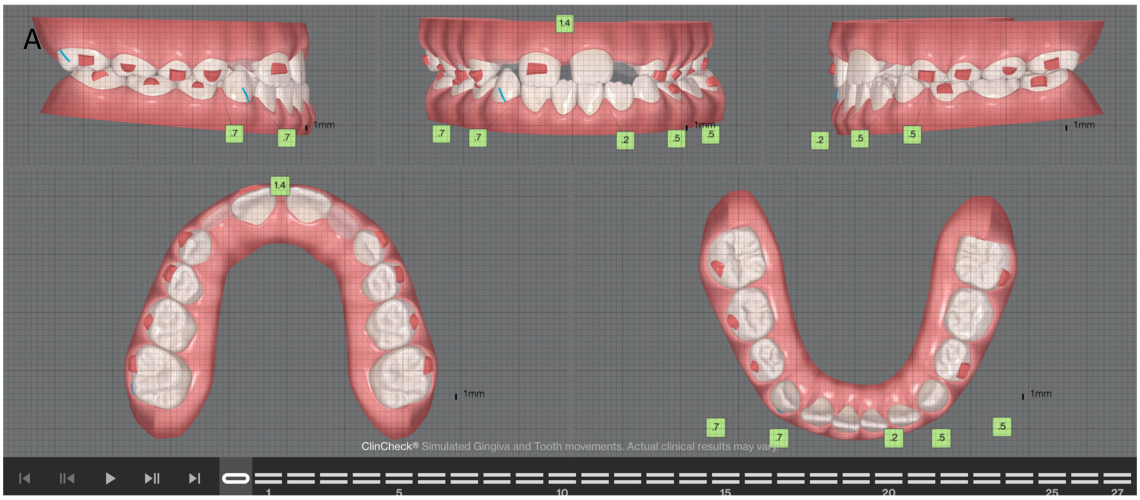
A
ClinCheck® Simulated Gingiva and Tooth movements. Actual clinical results may vary.

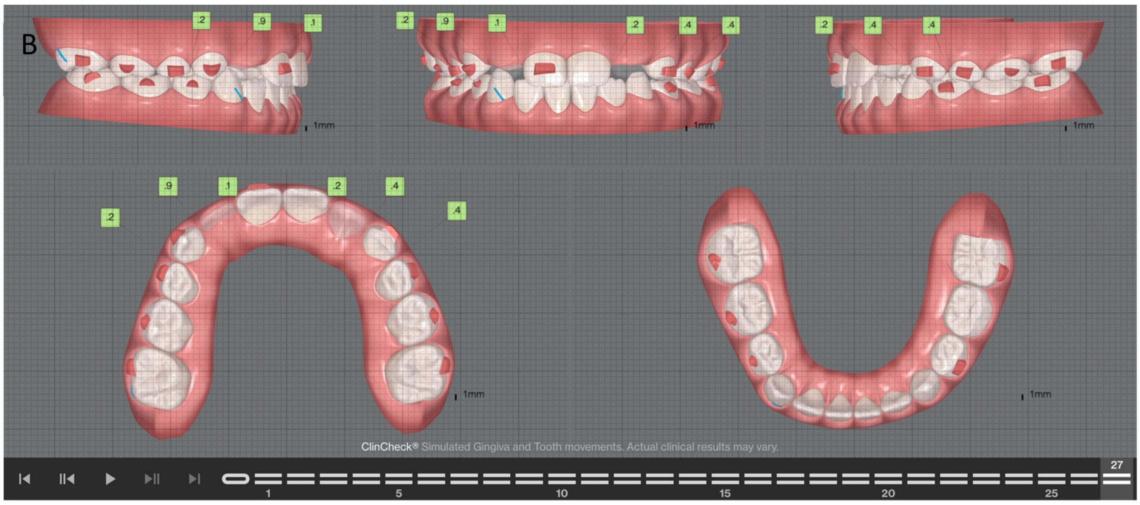
B
ClinCheck® Simulated Gingiva and Tooth movements. Actual clinical results may vary.

Case 20

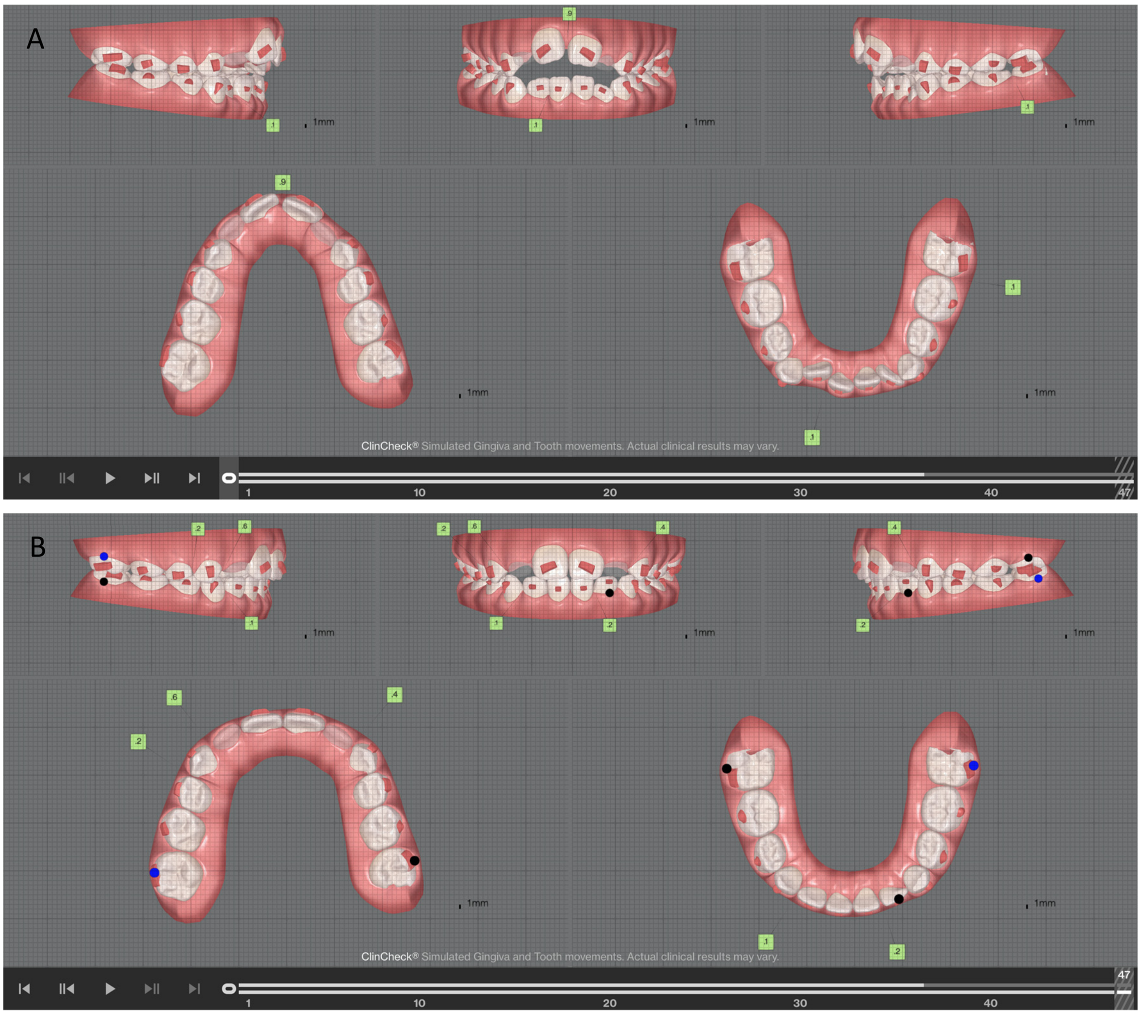
A
B
ClinCheck® Simulated Gingiva and Tooth movements. Actual clinical results may vary.

Case 21

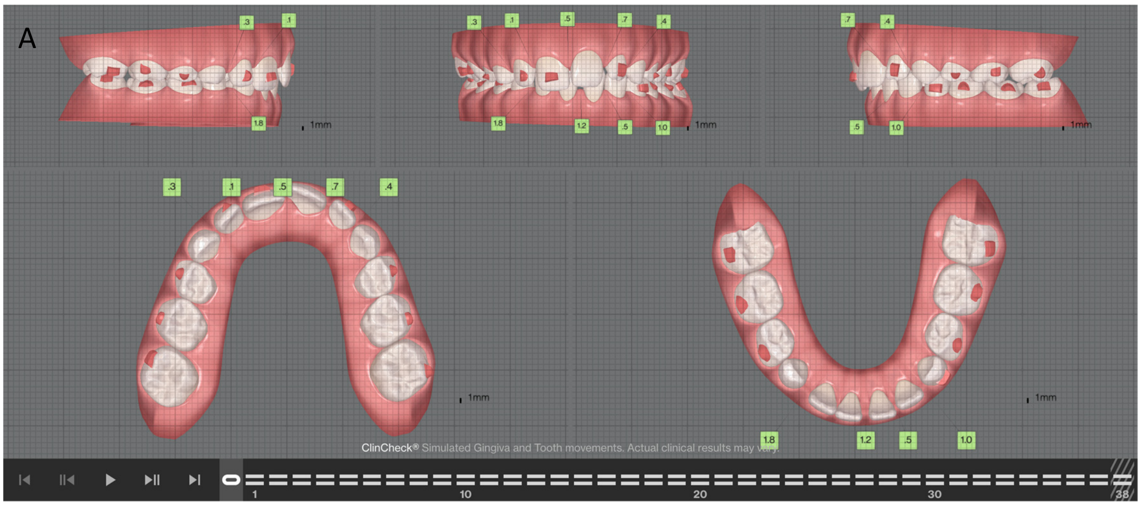
A
ClinCheck® Simulated Gingiva and Tooth movements. Actual clinical results may vary.

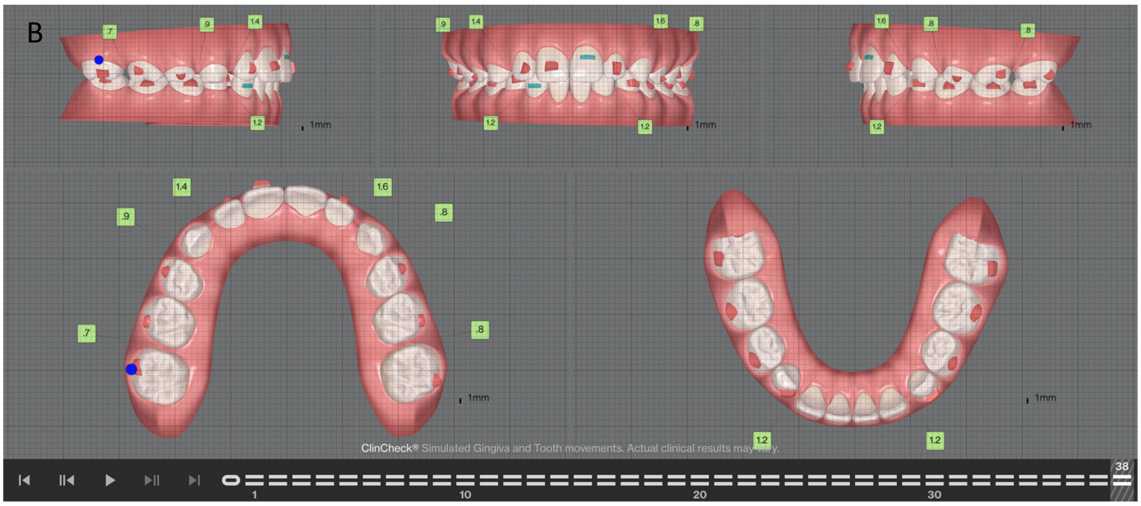
B
ClinCheck® Simulated Gingiva and Tooth movements. Actual clinical results may vary.

Case 22

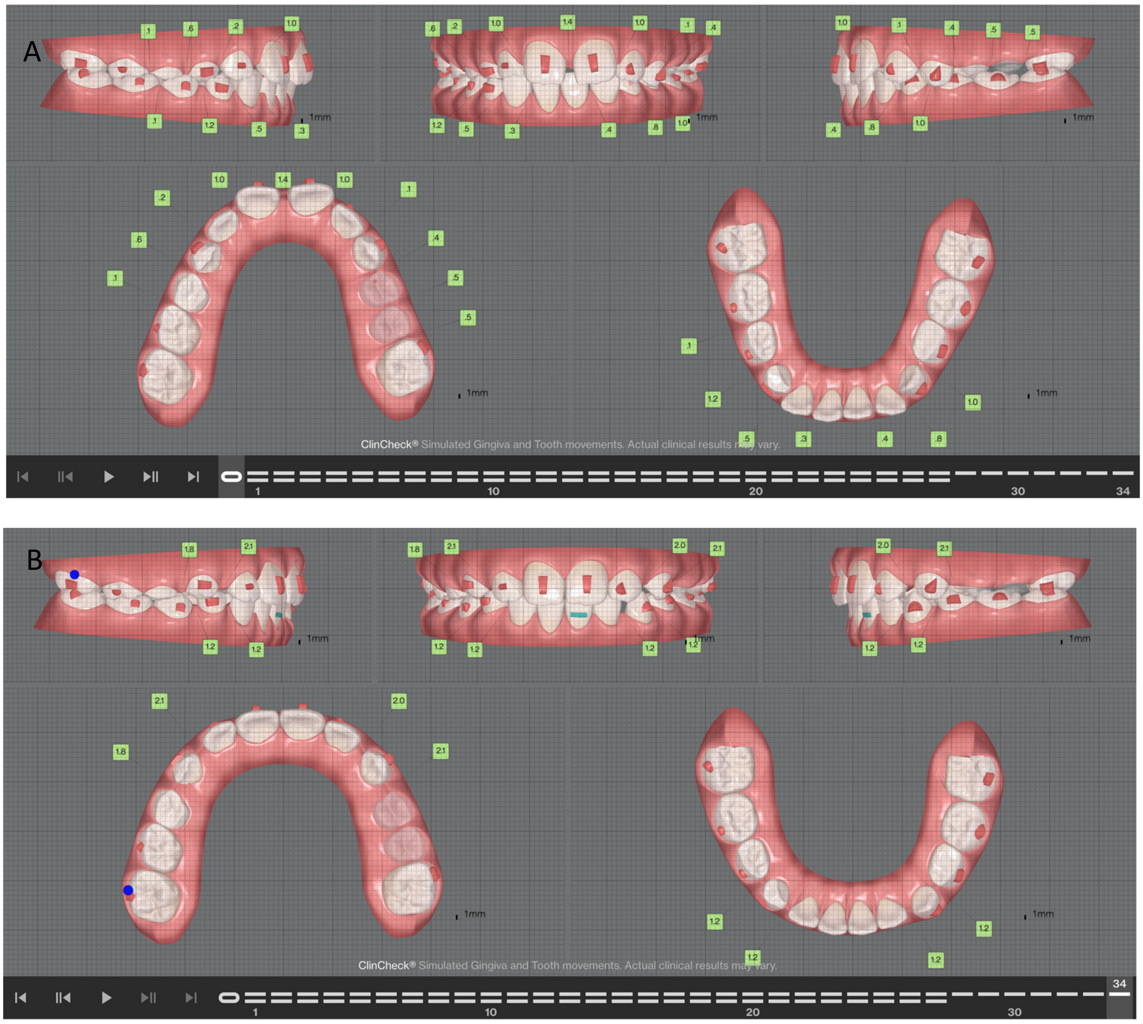

Case 23

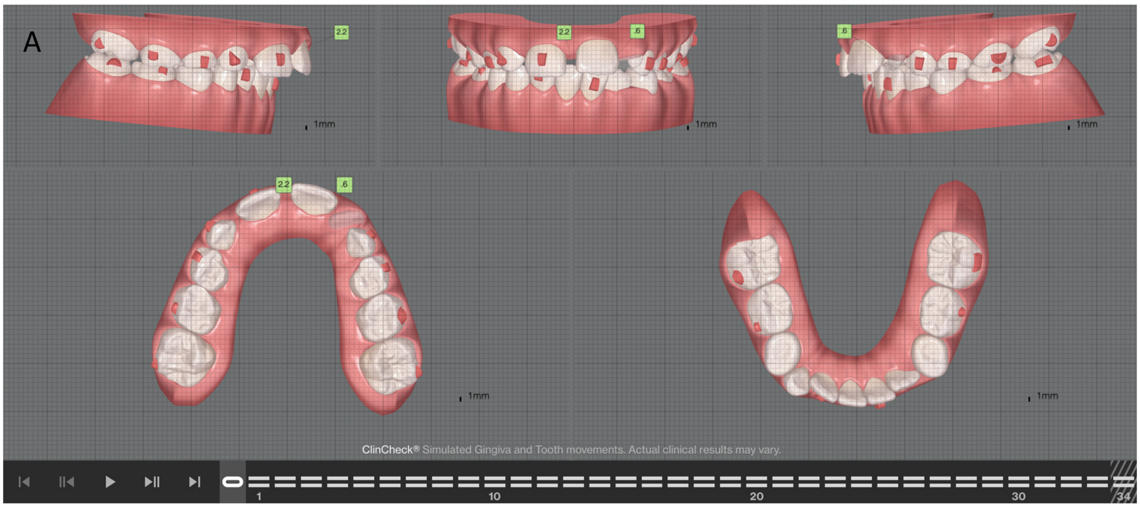
A
ClinCheck® Simulated Gingiva and Tooth movements. Actual clinical results may vary.

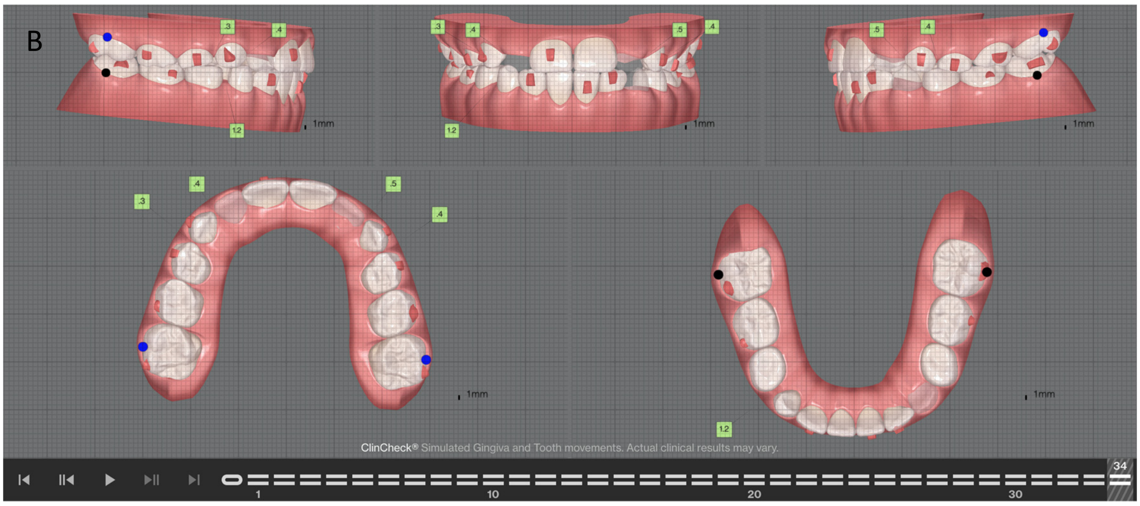
B
ClinCheck® Simulated Gingiva and Tooth movements. Actual clinical results may vary.
