# Supplementary material for: Interceptive Treatment with Invisalign® First in Moderate and Severe Cases: A Case Series
Source: Children (Basel). 2022 Aug 5;9(8):1176. doi: 10.3390/children9081176 (PMC9406487; doi:10.3390/children9081176)
Supplement: Supplementary file 1 [file children-09-01176-s001.zip › Pinho_et_al.-SupplementaryTables_final.pdf]

Supplementary Tables

Supplementary Table S1. Molar derotation planned on the first and last ClinCheck® scans of teeth 16 and 26, for nine patients.

Patient	Difficulty degree	Planned at the first ClinCheck®		Planned at the last ClinCheck®	
		Tooth 16	Tooth 26	Tooth 16	Tooth 26
Case 4	1	17.8° D	10.7° D	4.5º D	3.1º D
Case 5	1	16.6° D	17.8° D	6.1º D	2.4º D
Case 6	1	22.2° D	22.2° D	5.5º D	1.1º D
Case 11	1	24.0° D	20.9° D	0.3º D	4.8º D
Case 12	2	35.9° D	10.2° D	0.7ºD	0º
Case 18	1	10.4° D	17.7° D	0º	0º
Case 20	1	23.0° D	13.4° D	3.4º D	3.4º D
Case 21	1	25.5º D	14.8º D	2.5º D	0º
Case 22	1	10.9° D	16.6° D	0º	0º

(D) Distal Rotation.

Supplementary Table S2. Dentoalveolar expansion measured at the first and last ClinCheck® scans and planned at the last ClinCheck® between teeth 16 and 26, in 20 patients.

Patient	Difficulty Degree	Measured at the first ClinCheck®	Planned at the first ClinCheck®	Difference between measured and the first ClinCheck®	Measured at the last ClinCheck®	Difference between initially measured and the last ClinCheck®	Planned at the last ClinCheck®	Difference between measured and planned at the last ClinCheck®
Case 1	2	44.2	50.2	+6	47.5	+3.3	48.7	+1.2
Case 3	1	41.5	45.3	+3.8	44.6	+3.1	44.8	+0.2
Case 5	2	40.1	44.3	+4.2	43.7	+3.6	43.9	+0.2
Case 6	2	38.9	43.0	+4.1	40.6	+1.7	43.5	+2.9
Case 8	1	47.8	51.4	+3.6	51.4	+3.6	51.4	0
Case 9	2	45.5	49.6	+4.1	49.5	+4	49.5	0
Case 10	3	43.4	47.8	+4.4	48.2	+4.8	50.0	+1.8
Case 11	2	42.1	47.0	+4.9	47.0	+4.9	47.0	0
Case 12	2	41.9	47.0	+5.1	47.0	+5.1	47.0	0
Case 13	2	48.3	53.5	+5.1	53.5	+5.2	53.5	0
Case 14	1	43.4	46.1	+2.7	46.1	+2.7	46.1	0
Case 15	3	45.1	49.5	+4.4	49.5	+4.4	49.5	0
Case 16	3	44.4	48.4	+4.0	48.4	+4	48.4	0
Case 17	1	46.2	49.2	+3	49.5	+3.3	49.7	+0.2
Case 18	1	41.2	44.2	+3	43.8	+2.6	44.0	+0.2
Case 19	1	42.1	45.2	+3.1	46.4	+4.3	46.6	+0.2
Case 20	2	40.7	44.8	+4.1	40.9	+0.2	45.3	+4.4
Case 21	1	41.8	45.0	+3.2	45.3	+3.5	44.6	−0.7
Case 22	2	48.8	53.2	+4.4	53.2	+4.4	53.2	0
Case 23	3	43.6	47.7	+4.1	48.5	+4.9	48.9	+0.4

Supplementary Table S3. Space initially available and planned for the teeth. and space obtained at the end of treatment, for 16 patients.

Patient	Difficulty degree	Measured at the first ClinCheck® (available space)						Planned at the first ClinCheck®						Measured at the last ClinCheck® (obtained space)					
		Tooth						Tooth						Tooth					
		**12**	**15**	**22**	**32**	**33**	**43**	**12**	**15**	**22**	**32**	**33**	**43**	**12**	**15**	**22**	**32**	**33**	**43**
Case 1	3	2						7.5						7					
Case 2	2	4		4				7		6.5				6		6			
Case 3	1	4		5				6		6				6		6			
Case 4	2	4.5		4.5				7		7				6.5		6.5			
Case 5	3	4		3				7.5		7.5				6.5		7			
Case 6	1					4						6.5						6.5	
Case 8	1			5						7.5						7			
Case 9	2					2.5						7						7.5	
Case 10	2	3.5	2.5	6.5	4	1.5	3	7.5	8	8	6	6.5	6.5	7.5	8	8	6	6.5	6.5
Case 12	3		0						8						8				
Case 13	1			5.5						8						8			
Case 14	2	4		5.5				6.5		6.5				6.5		6.5			
Case 17	1	6						8						8					
Case 18	3	4.5		4.5			0	7		7			6.5	7		7			6.5
Case 20	1	5.5		5				7		7				7		7			
Case 23	3	1		4		0	5	7.5		7.5		7.5	6	7.5		7.5		7.5	6

Supplementary Table S4. Initial and final cephalometric measurements according for each parameter, for all patients.

Patient	Cephalometric parameters	Measured at the onset (1st)	Measured at the onset (2nd)	Difference (1st – 2nd)	Measured at the end (1st)	Measured at the end (2nd)	Difference (1st – 2nd)
Case 1	*ANB*	1.6	1.4	0.2	4	4	0
	FMA	33	29.7	3.3	28.3	28.1	0.2
	Overbite	2.7	2.7	0	3	3	0
	Overjet	0.7	0.7	0	3.7	3.7	0
Case 2	*ANB*	6.6	6.6	0	3.4	3.8	-0.4
	FMA	40	40.1	-0.1	30.6	31.4	-0.8
	Overbite	-4.1	-4.1	0	1.9	1.6	0.3
	Overjet	6	6	0	3.3	3.1	0.2
Case 3	*ANB*	7.8	7.9	0	3.1	3.1	0
	FMA	24.7	24.2	0.5	27.1	25.8	1.3
	Overbite	8.7	8.4	0.3	3.6	3.8	-0.2
	Overjet	5.3	5.3	0	4	4.3	0.3
Case 4	*ANB*	-1.5	-1.3	0	-2.9	-2.6	-0.3
	FMA	20	19.5	0.5	16.1	16.6	-0.5
	Overbite	2.1	2.1	0	3.8	4	-0.2
	Overjet	3.2	3.2	0	3.5	4.1	-0.6
Case 5	*ANB*	6.3	6.5	-0.2	4.8	4.8	0
	FMA	32.4	33.3	-0.9	27.3	26.8	0.5
	Overbite	-7	-7	0	1.3	1.3	0
	Overjet	1.5	1.5	0	2.4	2.4	0
Case 6	*ANB*	5.2	5.1	0.1	5.2	5.1	0.1
	FMA	20.9	21.7	-0.8	23.6	23.8	-0.2
	Overbite	3.4	3.3	0.1	4.2	4.2	0
	Overjet	4.9	4.1	0.8	4.9	4.9	0
Case 7	*ANB*	3	4.8	-1.8	3.4	3.5	-0.1
	FMA	26	25.6	0.4	31.8	32.2	-0.4
	Overbite	2	1.9	0.1	4	4	0
	Overjet	6.5	6.3	0.2	5.4	5.4	0
Case 8	*ANB*	0.9	1	-0.1	2.8	2.7	0.1
	FMA	27.6	28.1	-0.5	27.6	27.3	0.3
	Overbite	1.7	1.7	0	3.6	3.6	0
	Overjet	-1.6	-1.6	0	4.3	4.3	0
Case 9	*ANB*	3.3	3.3	0	2.7	2.7	0
	FMA	26	25.9	0.1	29.4	29.4	0
	Overbite	3.6	3.6	0	3.8	3.8	0
	Overjet	3.5	3.5	0	3.2	3.2	0
Case 10	*ANB*	4.6	4.8	-0.2	3.3	3.4	-0.1
	FMA	26.4	25.9	0.5	27	26.9	0.1
	Overbite	4.3	4.3	0	5.5	5.5	0
	Overjet	6.1	6.1	0	5.4	5.4	0

Case 11	*ANB*	6.4	6.4	0	3.2	3.3	-0.1
	FMA	36	35.6	0.4	36.5	36.5	0
	Overbite	0.9	0.9	0	2.3	2.3	0
	Overjet	6	6	0	3.2	3.2	0
Case 12	*ANB*	-1.6	-1.6	0	0.1	0.1	0
	FMA	33.9	33.6	0.3	33.2	32.8	0.4
	Overbite	0	-0.1	-0.1	2.7	2.7	0
	Overjet	0.3	0.2	0.1	3.4	3.4	0
Case 13	*ANB*	-0.3	0	-0.3	-0.4	-0.4	0
	FMA	27.4	28.3	-0.9	29.5	29	0.5
	Overbite	1.3	1.3	0	3.5	3.2	0.3
	Overjet	1.3	1.3	0	4.1	4.2	-0.1
Case 14	*ANB*	5.9	5.9	0	4.6	4.9	-0.3
	FMA	30.9	30.4	0.5	31.1	31.1	0
	Overbite	5.1	5.1	0	3.7	3.7	0
	Overjet	5.9	5.9	0	3.6	3.6	0
Case 15	*ANB*	6	6.2	-0.2	4.4	4.7	-0.3
	FMA	24.9	25.1	-0.2	26.6	25.8	0.8
	Overbite	5.5	5.5	0	5.2	5.2	0
	Overjet	4.2	4.2	0	4.4	4.4	0
Case 16	*ANB*	2.2	2.2	0	1.9	1.9	0
	FMA	23.2	23	0.2	24.7	24.1	0.6
	Overbite	0	0	0	2.6	2.6	0
	Overjet	0	0	0	2.5	2.5	0
Case 17	*ANB*	-0.1	0.1	-0.2	1.1	1.2	-0.1
	FMA	24.7	25.5	-0.8	27.1	27	0.1
	Overbite	-0.6	-0.6	0	2.8	2.8	0
	Overjet	3.2	3.2	0	4.1	4.1	0
Case 18	*ANB*	2.6	2.7	-0.1	1.5	1.6	-0.1
	FMA	30	29.9	0.1	32.5	32.5	0
	Overbite	4.5	4	0.5	3.1	3.1	0
	Overjet	7.2	7.6	-0.4	3.1	3.1	0
Case 19	*ANB*	2.8	3.2	-0.4	0.6	0.6	0
	FMA	29.1	28.4	0.7	28.3	28.6	-0.3
	Overbite	0.1	0.1	0	2.7	2.7	0
	Overjet	1.7	1.7	0	3	3	0
Case 20	*ANB*	7.4	7.3	0.1	4.6	4.6	0
	FMA	31.9	32	-0.1	34.5	35.3	-0.8
	Overbite	-2.9	-2.9	0	1.5	1.5	0
	Overjet	8.7	8.7	0	2.5	2.5	0
Case 21	*ANB*	5.1	5.1	0	3.7	3.7	0
	FMA	19.7	20.1	-0.3	19.4	19.4	0
	Overbite	4.9	4.9	0	3.2	3.2	0
	Overjet	3.4	3.4	0	2.5	2.5	0

Case 22	*ANB*	3.5	3.4	0.1	3.2	3.2	0
	FMA	22.9	23	-0.1	27.9	27.6	0.3
	Overbite	4	4	0	3	3	0
	Overjet	3.4	3.4	0	3.2	3.2	0
Case 23	*ANB*	6.2	6.6	-0.4	2.5	2.8	-0.3
	FMA	33	32.7	0.3	22.7	23	-0.3
	Overbite	3.2	3.2	0	5.7	5.7	0
	Overjet	9.4	9.4	0	3.3	3.3	0

Supplementary Table S5. Intra-operator error calculation for cephalometric measurements using the Dahlberg method.

ANB (Initial difference)	ANB (Final difference)	FMA (Initial difference)	FMA (Final difference)	Overbite (Initial difference)	Overbite (Final difference)	Overjet (Initial difference)	Overbite (Final difference)
0.04	0	10.89	0.04	0	0	0	0
0	0.16	0.01	0.64	0	0.09	0	0.04
0.01	0	0.25	1.69	0.09	0.04	0	0.09
0.04	0.09	0.25	0.25	0	0.04	0	0.36
0.04	0	0.81	0.25	0	0	0	0
0.01	0.01	0.64	0.04	0.01	0	0.64	0
3.24	0.01	0.16	0.16	0.01	0	0.04	0
0.01	0.01	0.25	0.09	0	0	0	0
0	0	0.01	0	0	0	0	0
0.04	0.01	0.25	0.01	0	0	0	0
0	0.01	0.16	0	0	0	0	0
0	0	0.09	0.16	0.01	0	0.01	0
0.09	0	0.81	0.25	0	0.09	0	0.01
0	0.09	0.25	0	0	0	0	0
0.04	0.09	0.04	0.64	0	0	0	0
0	0	0.04	0.36	0	0	0	0
0.04	0.01	0.64	0.01	0	0	0	0
0.01	0.01	0.01	0	0.25	0	0.16	0
0.16	0	0.49	0.09	0	0	0	0
0.01	0	0.01	0.64	0	0	0	0
0	0	0.16	0	0	0	0	0
0.01	0	0.01	0.09	0	0	0	0
0.16	0.09	0.09	0.09	0	0	0	0
0.29	0.11	0.60	0.35	0.09	0.08	0.14	0.10
